# Immune checkpoint inhibitors-induced pancreatitis: a systematic review and real-world pharmacovigilance analysis

**DOI:** 10.3389/fphar.2025.1426847

**Published:** 2025-03-19

**Authors:** Wei Fang, Huanping Wang, Xiaoran Zhang, Hongxia Zhu, Wei Yan, Yang Gao

**Affiliations:** ^1^ Department of Endocrinology, Chengdu Shuangliu Hospital of Traditional Chinese Medicine, Chengdu, China; ^2^ Department of Endocrinology, Hospital of Chengdu University of Traditional Chinese Medicine, Chengdu, China; ^3^ Laboratory of Ultrasound Medicine, West China Hospital, Sichuan University, Chengdu, China

**Keywords:** immune checkpoint inhibitors, pancreatitis, immune-related adverse event, immunotherapy, pharmacovigilance analysis

## Abstract

**Purpose:**

Immune checkpoint inhibitors-induced pancreatitis (ICIs-P) is an uncommon immune-related adverse event. The available evidence consists mostly of case reports, case series, and narrative reviews. This research focuses on the clinical characteristics and management options for ICIs-P to provide a practice-based global perspective on this disease.

**Methods:**

Five electronic databases were systematically reviewed to identify the relevant studies. Furthermore, we performed a disproportionality analysis utilizing OpenVigil 2.1 to interrogate the United States Food and Drug Administration’s Adverse Event Reporting System (FAERS) database.

**Results:**

A total of 61 patients from 58 studies were included in this study. Most patients with ICIs-P were males (60.7%). Most patients received anti-PD-1/PD-L1 monotherapy (78.7%) or anti-PD-1/PD-L1 monotherapy in conjunction with CTLA-4 blockade (19.7%). The median time from the initiation of immune checkpoint inhibitors treatment to pancreatitis was 108 days (range 52–278). Most cases were severe or life-threatening (G3–G4; 64.0%). Corticosteroids were administered to 73.8% of the patients during the treatment of pancreatitis. Regarding treatment outcomes, ICIs-P was reversible in most cases (83.6%), despite the 8.2% relapse and 8.2% deaths. We identified 606 reports of pancreatitis associated with ICIs in the FAERS database, with the greatest proportion of males (50.7%), 62.0% of PD-1 inhibitors, and 22.1% of all reports of death or life-threatening outcomes. Signals indicating pancreatitis were observed across all ICIs, with particular emphasis on Cemiplimab, Pembrolizumab and Nivolumab.

**Conclusion:**

By using a pharmacovigilance database, we discovered an elevated risk of pancreatitis following ICIs therapy, especially with PD-1 inhibitors. Meanwhile, risk factors for ICIs-P remain poorly understood, and diagnosis is challenging. Which may manifest as asymptomatic elevated pancreatic enzyme levels or clinical pancreatitis. Patients with pancreatitis symptoms should have their lipase and amylase levels and radiology evaluated. Diagnosis should be made by excluding other causes. Steroids are the cornerstone of ICIs-P treatment and slow dose reduction is recommended to reduce recurrence.

## 1 Introduction

Recently, immune checkpoint inhibitors (ICIs) have attracted considerable attention because of their remarkable efficacy. Immune checkpoints are the trigger points of immune system suppressive pathways and are mostly expressed on the surface of activated T lymphocytes, which can inhibit the killing effect of the immune system on target cells. Tumor cells evade the killing effects of the immune system by activating these inhibitory pathways. ICIs primarily include three types of cytotoxic T lymphocyte-associated antigen (CTLA-4) and programmed death receptor/ligand1 (PD-1/PD-L1) inhibitors (Postow et al., 2018). Patients may present with different clinical manifestations of related gland involvement, as ICIs may lead to excessive activation of T lymphocytes with serious side effects on the pituitary gland, thyroid gland, pancreas, and adrenal glands (Sznol et al., 2017). ICIs-P is a rare immune-related adverse event (irAEs) that causes a low quality of life and affects patient security (Jiang et al., 2018; Bai et al., 2021).

Although the incidence of ICIs-P is relatively low, its clinical manifestations exhibit considerable heterogeneity, ranging from asymptomatic biochemical abnormalities to severe acute pancreatitis (Nwankwo et al., 2024). The pathophysiological mechanisms underlying ICIs-P remain incompletely understood, with current evidence suggesting potential involvement of T cell-mediated autoimmune responses or dysregulation of immune tolerance (Fang et al., 2023). Furthermore, significant controversies persist regarding risk factors, diagnostic criteria, therapeutic strategies, and prognosis, due to the paucity of large-scale prospective studies (Brahmer et al., 2018; Thompson et al., 2019; Sayed et al., 2022). The existing literature predominantly consists of case reports and small retrospective studies (Kramer et al., 2023; Tanabe et al., 2024), with a notable scarcity of systematic reviews and real-world data analyses. In light of these limitations, this study aims to comprehensively evaluate the clinical characteristics, risk factors, and therapeutic strategies of ICIs-P through systematic review of case reports and analysis of real-world pharmacovigilance data. By integrating existing evidence with real-world data, we anticipate providing clinicians with more comprehensive diagnostic and therapeutic references, thereby optimizing the management of ICIs-P.

## 2 Materials and methods

### 2.1 Systematic review

#### 2.1.1 Search strategy

This review was designed in accordance with the PRISMA guidelines. PubMed, Web of Science, Cochrane Library, and EMBASE databases were retrieved from the inception date to February 2025 using the literature search strategy reported in Supplementary Table S1 (Supplementary Information 1). The references of the included studies were manually searched to retrieve additional eligible studies. Only English and Chinese publications were included in this analysis.

#### 2.1.2 Selection criteria

The following study types were included: case reports, case series, observational studies, randomized controlled trials (RCTs), review articles, letters, and correspondence involving relevant cases. Meta-analyses, duplicate cases, review articles lacking patient information, conference abstracts, and animal studies were also excluded. The inclusion criteria were as follows:1) studies containing individual case reports or case series and 2) patients with confirmed ICIs-P association. The diagnosis of ICIs-P was based on the National Comprehensive Cancer Network (NCCN) classification criteria or the physician’s opinion. If the authors did not specify the NCCN classification, we inferred it based on the clinical information (Thompson et al., 2019). The specific diagnostic criteria are listed in Table 1.

**TABLE 1:** Diagnostic criteria for immune checkpoint inhibitors-induced pancreatitis.

① A clear history of use of immune checkpoint inhibitors
② At least two of the following three criteria were met
(1) Persistent pain in the upper abdomen; (2) elevated serum lipase/amylase levels (at least three times the upper normal limit); and (3) characteristic findings of acute pancreatitis on abdominal imaging
③ Other causes of acute pancreatitis were excluded

#### 2.1.3 Data extraction

Reference identification and data collection were performed individually by two reviewers (Y.G. and W.F.) following the established criteria and data collection forms. Any disagreements were resolved through joint negotiations, and if a consensus could not be reached, an adjudication was performed by a third researcher (W.Y.). The titles and abstracts of the retrieved publications were screened to identify potential articles, and full texts were screened. The purpose of this review was to compare variables between studies. To obtain maximum information, we did not use quality assessment as an article inclusion criterion. Information from the included studies was extracted as follows: first author; publication year; age; sex; race; tumor type; checkpoint inhibitors treatment; number of cycles or days of treatment; symptoms at onset; IgG4 antibody; relevant prior medical history; presence of diabetes; glucose, glycated hemoglobin, lipase, and amylase levels; imaging; ICIs management; other irAEs; and outcomes.

### 2.2 Pharmacovigilance analysis

#### 2.2.1 Data sources and collection

FAERS is a database used for post-marketing monitoring of all drugs and therapeutic biological products approved by Food and Drug Administration (FDA). OpenVigil 2.1 is a publicly available tool for extracting FAERS-related data (http://openvigil.sourceforge.net/). In this study, OpenVigil 2.1 was used to obtain adverse events data in FAERS from the time of initial FDA approval to 30 September 2024, and drug names were standardized according to Drugbank and Drugs@FDA.

The search for ICIs included its generic name and brand names (Table 2). Utilizing MedDRA version 26.0, we identified 25 preferred terms (PTs) listed in Supplementary Table F1 (Supplementary Information 2) to systematically collect cases associated with “acute pancreatitis” (Standardized MedDRA Queries (SMQ): 20000022) and closely related clinical conditions (Guo et al., 2024). We gathered detailed clinical information for each adverse event report, encompassing data such as outcomes, medication names, role codes, dosages, indications, events, genders, reporter countries, and ages. For the collected data, first, we selected only the reports listed as the primary suspected drug and excluded the remaining reports. Second, in adherence to the guidelines of FDA, our study implemented a rigorous process to identify and eliminate duplicate reports. The data filtering procedure employed in this study is detailed in Supplementary Figure F1 (Supplementary Information 2).

**TABLE 2:** List of ICIs marketed in the United States and dates of FDA approval.

Effect target	Drug name	Brand names	FDA Approval time
PD-1			
	Pembrolizumab	*Keytruda*	September 2014
	Nivolumab	*Opdivo, Opdualag*	December 2014
	Cemiplimab	*Libtayo*	September 2018
	Dostarlimab	*Jemperli*	April 2021
	Toripalimab	*Loqtorzi*	October 2023
	Retifanlimab	*Zynyz*	March 2023
PD-L1			
	Atezolizumab	*Tecentriq,Tecentriq,Hybreza*	May 2016
	Durvalumab	*Imfinzi*	May 2017
	Avelumab	*Bavencio*	March 2017
CTLA-4			
	Ipilimumab	*Yervoy*	March 2011
	Tremelimumab	*Imjudo*	October 2022

#### 2.2.2 Signal mining

In this research, four widely utilized disproportionality analysis techniques were implemented: reporting odds ratio (ROR), proportional reporting ratio (PRR), Bayesian confidence propagation neural network (BCPNN), and multi-item gamma Poisson shrinker (MGPS) (Wang et al., 2024). ROR and PRR quantify the relationship between actual and anticipated reporting frequencies, where elevated values suggest a more pronounced drug-adverse event (AE) correlation. Both BCPNN and MGPS use Bayesian statistical approaches in their computations. Notably, MGPS yields more consistent results than ROR, effectively decreasing the likelihood of false positive outcomes. Simultaneously, BCPNN’s Information Component (IC) serves as an indicator of the intensity of drug-AE signal associations (Wu et al., 2024; Noguchi et al., 2021). The integration of these four methodologies in our investigation significantly enhances the reliability of drug-AE signal detection while substantially reducing the occurrence of false positive results. The equations and criteria for these algorithms are detailed in Supplementary Table F2 (Supplementary Information 2). If any of the four algorithms met the predefined criteria, a positive signal of pancreatitis was identified (Guo et al., 2024).

### 2.3 Statistical analysis

SPSS v.22.0 (SPSS Inc., Chicago, IL, USA) was used for all the statistical analyses. Categorical variables are expressed as numbers and percentages, and continuous variables are expressed as medians and interquartile ranges (IQR).

## 3 Results

### 3.1 Literature search results

**FIGURE 1:**
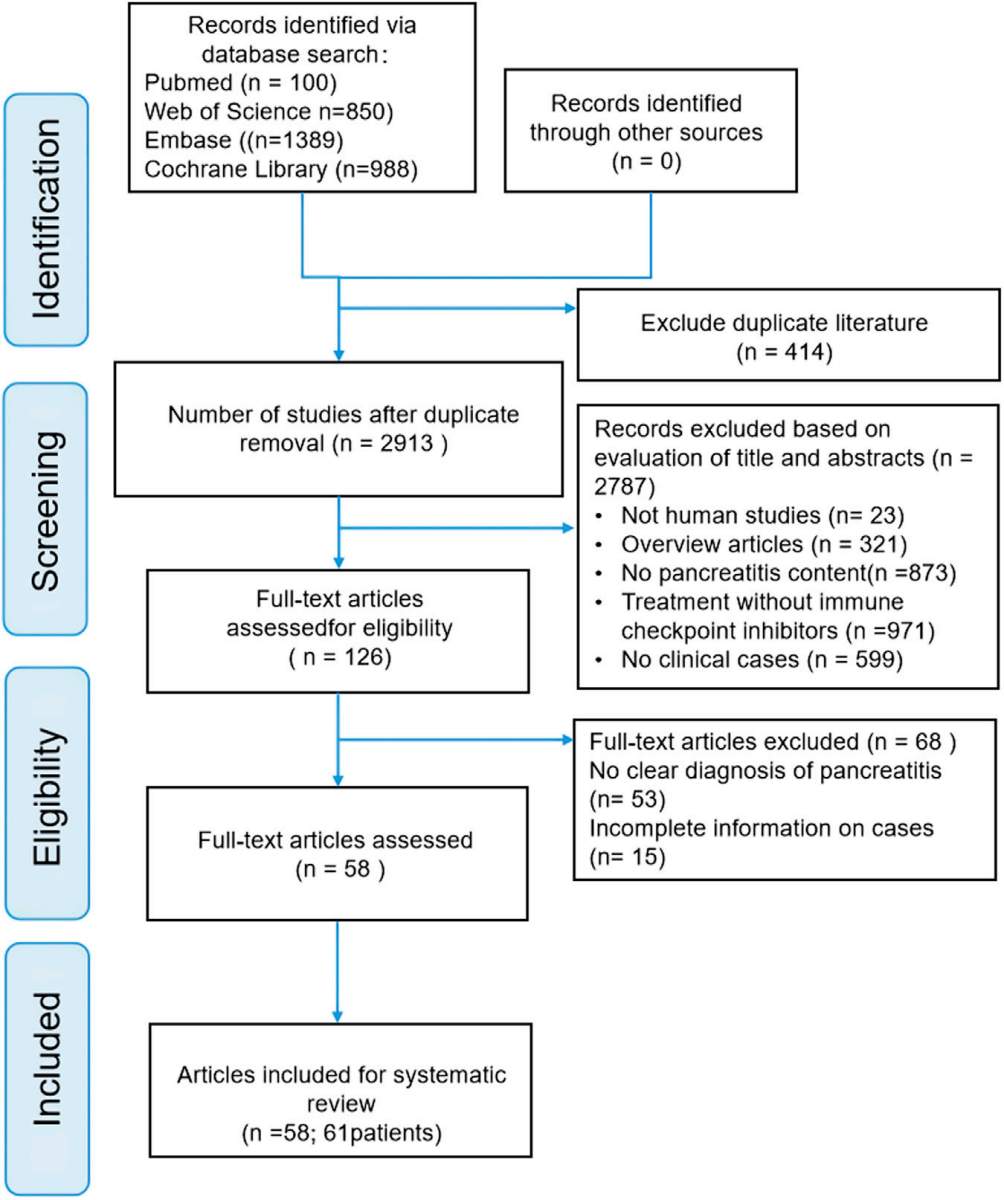
Flow chart of study selection.

Our literature search found 3,327 articles in the selected databases, and no article was found in gray literature. First, 414 duplicate articles were excluded from analysis. Then of 2,787 references were excluded by browsing titles and abstracts for meta-analyses, reviews, absence of clinical cases, and irrelevant literature. Of the remaining 126 records, 68 were further excluded after reading the complete text for the following reasons: no precise diagnosis of pancreatitis (n = 53) and incomplete cases (n = 15). Ultimately, 58 articles involving 61 patients were included in this review. The screening process is illustrated in Figure 1. Supplementary Table S2 (Supplementary Information 1) shows basic information on the 58 publications.

### 3.2 Patient characteristics

A summary of the main characteristics of the 61 patients is presented in Table 3. Most patients were male (37/61, 60.7%), and the median age at ICIs-P diagnosis was 58 years (range, 23–82 years). The predominant tumor types were melanoma (18/61, 29.5%) and NSCLC (16/61, 26.2%). The most commonly used immune checkpoint drugs for monotherapy were pembrolizumab (23/61, 37.7%) and nivolumab (17/61, 27.9%), while nivolumab and ipilimumab were the most commonly used combination drugs (7/61, 11.5%). Blocking the PD-1/PD-L pathway was observed in 78.7% (48/61) of the cases. The median time to onset of pancreatitis after the start of ICIs was 108 (range, 52–278) days, but there were some cases of early toxicity occurring on day 1 of treatment (Jiang et al., 2018)or late toxicity occurring after 1 year of therapy with ICIs (Bachiller et al., 2020; Kakuwa et al., 2020; Yilmaz and Baran, 2022) or even after the end of treatment (Capurso et al., 2018; Dehghani et al., 2018; Wright et al., 2021).

**TABLE 3:** Summary results on the characteristics of patients with ICIs-P.

Characteristic	All cases (N = 61)
Age, years	
Median (range)	58 (23–82)
Gender	N (%)
Male/Female, N (%)	37(60.7)/24(39.3)
Tumor type	N (%)
Melanoma	18 (29.5)
NSCLC	16 (26.2)
RCC	6 (9.8)
UC	2 (3.3)
metrocarcinoma	2 (3.3)
Other Tumor	17 (27.9)
Agent(ICIs)	N (%)
Pembrolizumab	23 (37.7)
Nivolumab	17 (27.9)
Toripalimab	3 (4.9)
Atezolizumab	2 (3.3)
Nivolumab + Ipilimumab	7 (11.5)
Ipilimumab + Pembrolizumab	4 (6.5)
Pembrolizumab + bevacizumab	1 (1.6)
Other ICIs (Frequency only 1 time)	4 (6.5)
ICIs type	N (%)
Anti-PD-1/L1	48 (78.7)
Anti-CTLA-4	1 (1.6)
Combination	12 (19.7)
The median time of onset, day (min-max) [IQR]	108 (1–1,020) [52–278]
Symptoms	N (%)^#^
Typical symptoms of pancreatitis	46 (78.0)
Asymptomatic	9 (15.2)
Nonspecific symptoms	4 (6.8)
Elevation of serum amylase or lipase	N (%)
Yes/No	55(90.2)/6(9.8)
Imaging findings of pancreatitis	N (%)^#^
Yes/No	53(91.4)/5(8.6)
Grading of pancreatitis	N (%)
G3-G4	39 (64.0)
G2	15 (24.5)
G1	7 (11.5)
Other immune-related adverse events	N (%)*
Colitis	8 (12.3)
Hepatobiliary injury	12 (18.5)
Dysthyroidism (hyper/hypo)	5 (7.6)
Gastritis	2 (3.1)
Hyperlipemia	3 (4.6)
Other irAEs	6 (9.2)
None	29 (44.6)
Treatment of pancreatitis	N (%)
Intravenous fluids	6 (9.8)
Steroids/Steroids and other treatments	
For pancreatitis only	27 (44.3)
For other reasons also	18 (29.5)
Discontinuation of ICIs therapy only	2 (3.3)
Other	8 (13.1)
Management of ICIs	N (%)^#^
Permanently discontinued	30 (58.8)
Temporarily discontinued, then restarted	7 (13.7)
Continued	3 (5.9)
Treatment already completed at the onset	11 (21.6)
Outcome	N (%)
Improvement	51 (83.6)
Death	5 (8.2)
Recurrence	5 (8.2)

Abbreviations: ^#^Some articles are not available; *Some cases had multiple adverse reactions; ICIs-P, Immune checkpoint inhibitors-induced pancreatitis; N,number; NSCLC, non-small cell lung carcinoma; RCC, renal cell carcinoma; UC, urothelial carcinoma; PD-1, programmed cell death protein 1; PD-L1, programmed death ligand 1; CTLA-4, cytotoxic T lymphocyte antigen 4; IQR, interquartile range.

At the onset of pancreatitis,78.0% (46/59) of the patients presented with typical pancreatitis symptoms, such as abdominal pain and vomiting. Additionally, 6.8% (4/59) presented with atypical pancreatitis symptoms, such as weakness and thirst, and 15.2% (9/59) had no symptoms. Regarding ancillary tests, 90.2% (55/61) of the patients showed varying degrees of lipase or amylase elevation, and 91.4% (53/58) exhibited typical imaging manifestations of pancreatitis. Most patients with ICIs-P (39/61, 64.0%) had severe (G3) or life-threatening (G4) disease. The most common irAEs were colitis (8/65, 12.3%), hepatobiliary injury (12/65, 18.5%), and dysthyroidism (5/65, 7.6%).

Overall, corticosteroids were used in 73.8% (45/61) of the cases during the treatment of pancreatitis. Common corticosteroids include prednisone, prednisolone, and methylprednisolone. In a few mild cases, only rehydration therapy was used (6/61, 9.8%), and ICIs were discontinued (2/61, 3.3%). Regarding ICIs management, 72.5% (37/51) of patients discontinued ICIs during the treatment of pancreatitis, 58.8% (30/51) Permanently discontinued ICIs, and 13.7% (7/51) continued ICIs. In terms of treatment outcomes, although ICIs-P was largely reversible, with an improvement rate of 83.6% (51/61), 8.2% (5/61) of patients had a relapse, and 8.2% (5/61) of patients had an associated death. Notably, seven patients who were reintroduced to ICIs did not experience pancreatitis recurrence, and five recurrences occurred during steroid tapering.

Additionally, among the 61 patients, we identified four patients with pancreatitis and diabetes mellitus (Supplementary Table S3, Supplementary Information 1). All patients had a history of nivolumab treatment except one who was treated with Toripalimab, and elevated pancreatic enzyme levels and imaging changes were typically observed during all episodes of pancreatitis. One patient with a concurrent onset of pancreatitis and diabetes mellitus had improved outcomes without steroid treatment (Fang et al., 2023).

### 3.3 Descriptive analysis from FAERS

The FAERS database documented a total of 606 cases of ICIs-P between March 2011 and September 2024. Among the reported cases, PD-1 inhibitors accounted for 62.0% (376/606), PD-L1 inhibitors for 23.3% (141/606), and CTLA-4 inhibitors for 14.7% (89/606). The demographic and clinical characteristics of all ICIs-associated pancreatitis cases are detailed in Supplementary Table F3 (Supplementary Information 2). Specifically, 179 cases were associated with Nivolumab (179/606, 29.5%), 189 with Pembrolizumab (189/606, 31.2%), 106 with Atezolizumab (106/606, 17.5%), and 88 with Ipilimumab (88/606, 14.5%). Males (307/606, 50.7%) were more frequently affected than females (217/606, 35.8%), with the majority of cases occurring in the 61–80 age group (260/606, 42.9%). Notably, only two cases involving Pembrolizumab were reported in children or adolescents (2/606, 1.1%). Hospitalization was the most common outcome (231/606, 38.1%), while death or life-threatening outcomes accounted for 22.1% (134/606) of all reports. The top three reporting countries were Japan (38.9%, 236/606), the United States (30.7%, 186/606), and France (6.6%, 40/606).

### 3.4 Signal values associated with different ICIs

The identification of pancreatitis event signals associated with all ICIs was conducted following the criteria set by the four algorithms, and the corresponding results are detailed in Table 4. Apart from Dostarlimab (2/606), Toripalimab (2/606), Retifanlimab (0/606), and Tremelimumab (1/606), where the small number of cases may introduce uncertainty in the results and further research is needed for validation, the remaining ICIs satisfied all four criteria. Notably, among all ICIs, Cemiplimab demonstrated the strongest association with ICIs-P, as evidenced by an information component (IC) of 7.20 (IC025 3.54), a reporting odds ratio (ROR) of 151.20 (95% CI 74.39–307.36), a proportional reporting ratio (PRR) of 150.63 (χ^2^ 581.12), and an empirical Bayes geometric mean (EBGM) of 147.25 (EBGM05 76.96). Following Cemiplimab, Atezolizumab, Avelumab, Ipilimumab, Pembrolizumab, Nivolumab, and Durvalumab exhibited progressively lower values.

**TABLE 4:** Associations of immune checkpoint inhibitors with pancreatitis.

ICIs	N	ROR (95% CI)	PRR (χ2)	IC (IC025)	EBGM (EBGM05)
PD-1					
Pembrolizumab	189	2.30 (2.08.2.55)	2.30 (139.97)	1.20 (1.08)	2.29 (2.09)
Nivolumab	179	1.91 (1.73.2.12)	1.91 (79.83)	0.93 (0.84)	1.91 (1.74)
Cemiplimab	4	151.20 (74.39,307.36)	150.63 (581.12)	7.20 (3.54)	147.25 (76.96)
Dostarlimab	2	2.11 (0.78.5.68)	2.11 (1.16)	1.07 (0.40)	2.11 (0.85)
Toripalimab	2	1.64 (0.61.4.43)	1.64 (0.50)	0.71 (0.26)	1.64 (0.66)
Retifanlimab*	-	-	-	-	-
PD-L1					
Atezolizumab	106	3.05 (2.67.3.49)	3.05 (150.87)	1.60 (1.40)	3.04 (2.69)
Durvalumab	27	1.86 (1.44.2.42)	1.86 (11.59)	0.90 (0.69)	1.86 (1.47)
Avelumab	8	3.04 (1.79.5.17)	3.04 (9.58)	1.60 (0.94)	3.04 (1.87)
CTLA-4					
Ipilimumab	88	2.49 (2.15.2.89)	2.49 (78.37)	1.31 (1.13)	2.48 (2.17)
Tremelimumab	1	220.86 (53.76,907.30)	218.75 (214.86)	7.76 (1.89)	216.84 (59.54)

Abbreviations: *No target incident reported; N,number; ICIs, Immune checkpoint inhibitors; ROR, reporting odds ratio; CI, confidence interval; PRR, proportional reporting ratio; χ2, chi-squared; IC, information component; EBGM, empirical Bayes geometric mean;PD-1, programmed cell death protein 1; PD-L1, programmed death-ligand 1; CTLA-4, cytotoxic T lymphocyte antigen 4.

## 4 Discussion

### 4.1 Clinical features

From March 2011 to September 2024, the FAERS database documented 606 cases of immune checkpoint inhibitor-associated pancreatitis. Both pharmacovigilance analyses and retrospective case series revealed a male predominance, with a peak incidence in the 61–80 age group, consistent with the typical cancer onset age range and prior studies (Hori et al., 2024). Notably, only two cases involved children or adolescents, suggesting a lower incidence of ICIs-P in younger populations, which may be attributed to the infrequent use of immune checkpoint inhibitors in pediatric patients. PD-1 inhibitors were identified as the primary causative agents of ICIs-P, with pembrolizumab and nivolumab accounting for the highest proportions. A similar phenomenon was observed in case reviews. This distribution likely reflects the widespread clinical application of PD-1 inhibitors, particularly as first-line treatment options for various cancers (Kramer et al., 2023; Nwankwo et al., 2024). It is noteworthy that all immune checkpoint inhibitors exhibited positive signals for pancreatitis adverse events in the disproportionality analysis, although results for some drugs may be uncertain owing to limited case numbers. Statistically significant associations with pancreatitis were observed for pembrolizumab, nivolumab, atezolizumab, ipilimumab, cemiplimab, and tremelimumab. Retrospective case series indicated that melanoma and non-small cell lung cancer were the predominant tumor types, with a median time to pancreatitis onset of 108 days following ICIs treatment, consistent with previous findings (Sakaguchi et al., 2024). Typical pancreatitis symptoms were present in 78.0% of patients, while 15.2% were asymptomatic. Nearly all patients exhibited elevated lipase or amylase levels, and 64.0% experienced severe (Grade 3) or life-threatening (Grade 4) ICIs-P. Pharmacovigilance analyses also revealed that death or life-threatening outcomes accounted for 22.1% of cases. These findings suggest that ICIs-P represents a potentially life-threatening immune-related adverse event, warranting heightened vigilance among clinicians using immune checkpoint inhibitors, particularly in high-risk patients.

### 4.2 Controversial risk factors

The exact prevalence of ICIs-P remains unclear, with reported rates ranging from 0.3% to 14% (Michot et al., 2018; Su et al., 2018; George et al., 2019). This wide range may be due to the heterogeneity generated by the different severities of the cases and potential risk factors in these studies. Regarding patient characteristics, our study showed that male sex and melanoma appeared to increase the risk of developing ICIs-P. This finding agrees with the results of previous publications (Zhang et al., 2022). In clinical practice, ICIs should be used more cautiously in female patients because they are more likely to develop autoimmune disorders than male patients (Quintero et al., 2012; Conforti et al., 2018). Several researchers have analyzed the correlation between sex and pancreatic AEs and concluded that there is no noticeable discrepancy in irAEs between male and female patients (Jing et al., 2021; Ma et al., 2021; Zhang et al., 2022). In addition, our review indicated that ICIs-P patients aged <65 years were reported more frequently than those aged ≥65 yrs. Meanwhile, pharmacovigilance analysis also indicates that the age distribution of ICIs-P mainly concentrates in the range of 61–80 years old. However, the effect of age on ICIs-P is controversial, as some studies have reported a slightly higher prevalence of irAEs in older patients (Baldini et al., 2020), across the spectrum of irAEs (Paderi et al., 2021). Other studies have shown that age was not associated with irAEs (Gomes et al., 2021; Noseda et al., 2021). Therefore, future studies should focus on the sex and age disparities in patients with irAEs.

Notably, the strongest association between anti-PD-1 and ICIs-P among all the monotherapies was observed in our analysis, which is consistent with the results of two previous studies (Reese et al., 2020; Zhang et al., 2022). However, the association between ICIs and pancreatitis remains unclear. Several studies have shown that the prevalence of pancreatitis in ICIs therapy using anti-CTLA4 alone or in conjunction with nivolumab and ipilimumab is higher than that using PD-1/PD-L1 alone (Su et al., 2018; George et al., 2019; Bai et al., 2021). Therefore, prospective studies are required to investigate the exact association between pancreatitis and various ICIs therapies.

### 4.3 Challenging diagnoses

Accurate diagnosis of ICIs-P remains challenging because its clinical presentation can be insidious. The determination of acute pancreatitis requires at least two of the following characteristics: clinical symptoms in the abdomen, elevated pancreatic enzymes (serum lipase/amylase levels at least three times the normal value), and imaging findings of pancreatitis (Liu et al., 2021), such as Computed Tomography (CT), Magnetic Resonance Imaging (MRI), and Positron Emission Tomography/Computed Tomography (PET/CT), demonstrating any of the following: (1) new focal or diffuse pancreatic enlargement; (2) diminished attenuation, surrounding fat stranding, and no suspicious metastases; and (3) diffuse enhanced F-deoxyglucose uptake (Alessandrino et al., 2019; Das et al., 2020). Currently, the CTCAE 5.0 (Freites-Martinez et al., 2021) and the NCCN (Thompson et al., 2019) provide insignificantly different grading criteria for ICIs-related pancreatic injury severity according to asymptomatic elevated pancreatic enzymes and pancreatitis.

However, this rare ICIs-P event can be observed as a common clinical symptom of acute pancreatitis or as an asymptomatic incidental finding (Chandler et al., 2021; Tanaka et al., 2021). Increased serum amylase and/or lipase levels in ICIs-P can also indicate asymptomatic or radiological abnormalities, and elevated pancreatic enzymes are confounding factors (Abu-Sbeih et al., 2019). Therefore, the NCCN guidelines do not recommend routine testing of pancreatic enzymes at baseline or during ICIs treatment. It follows that a ruled-out diagnosis establishes an ICIs-P diagnosis. The initial examination includes a comprehensive assessment of other causes, such as alcohol, gallstones, hypertriglyceridemia, drugs, viruses, genetic susceptibility, tumors, and anatomical variants (Grover et al., 2018).

In addition, Abu-Sbeih et al. reported that ICIs-Ps occurred frequently in patients who presented with additional irAEs (Abu-Sbeih et al., 2019). Thus, we suggest measuring lipase levels in patients with adverse events unrelated to the pancreas. Our findings showed that 55.4% of the patients treated with ICIs-P had other irAEs. Furthermore, some patients with elevated pancreatic enzyme levels can have pancreatitis detectable on abdominal imaging despite the absence of symptoms (Saito et al., 2019; Chandler et al., 2021; Yazaki et al., 2022). Our data showed that most patients with ICIs-P presented with the typical symptoms of pancreatitis (78.0%), abnormal laboratory findings (90.2%), and imaging abnormalities (91.4%). This could be because we excluded patients with elevated pancreatic enzyme levels who did not meet the diagnostic criteria for pancreatitis. Accordingly, imaging is recommended for patients treated with ICIs-P when elevated pancreatic enzymes are found to avoid delaying diagnosis. However, differential diagnosis remains challenging because the imaging features of ICIs-P are similar to those of autoimmune pancreatitis (AIP). Some scholars have defined ICIs-P as a type 3 AIP and have suggested that its diagnosis could be based on the AIP criteria (Shimosegawa et al., 2011; Okazaki et al., 2014). We summarized the available research evidence on Table 5 (Das et al., 2019; Ofuji et al., 2021; Yamamoto et al., 2021; Sayed et al., 2022; Zen, 2022).

**TABLE 5:** Comparison between three types of autoimmune pancreatitis.

Subtype of AIP	Type 1	Type 2	Type 3
Other nomenclatures	AIP without GEL IgG4-related LPSP	AIP with GEL IgG4-unrelated IDCP	Immune checkpoint inhibitors-induced pancreatitis
Prevalence	Asia > USA > EU	EU > USA > Asia	Unclear
Age	Old (60, typically >40)	Young (30, including children)	Aged (60, typically <65)
Gender	male >> female	male = female (NS)	male = female (NS)
Symptoms			
Obstructive jaundice	Often	Often	Rare
Abdominal pain	Rare	Common	Common
Pancreas swelling	Common	Common	Common
Serum levels of IgG4	Elevated (80%–90%)	Normal (10%)	Normal
Histology	Lymphoplasmacytic periductal infiltration	Granulocytic duct wall infiltration	T-lymphocyte and neutrophil infiltration
		Granulocyte epithelial lesions (GEL)	Increased CD8^+^/CD4^+^ T-cell ratio
			Acinar-ductal metaplasia
			Fibrosis
IgG4 + cells	++	- or +	- or +
Other organ Involvement (OOI)	Sclerosing cholangitis	Unrelated with OOI	Unrelated with OOI
	Sclerosing sialadenitis		
	Retroperitoneal fibrosis		
	others		
Ulcerative Colitis	Rare	Often	Common
Steroid	Responsive	Responsive	Partially responsive
Recurrence	High rate	Rare	Rare

Abbreviations: AIP, autoimmune pancreatitis; GEL, granulocyte epithelial lesions; IgG4, serum immunoglobulin G subtype 4; LPSP, lymphoplasmacytic sclerosing pancreatitis; IDCP, lidiopathic duct centric pancreatitis; EU, european union; USA, united states of america.

### 4.4 Treatment and management

Owing to the rarity and insufficient evidence of ICIs-P, only the NCCN has proposed management guidelines for ICIs-P based on its severity (Thompson et al., 2019). Other guidelines provide no opinion or limited advice regarding the treatment of this irAEs (Brahmer et al., 2018; Haanen et al., 2018; Brahmer et al., 2021). The NCCN guidelines do not recommend intervention for grade G1 pancreatitis (mild or asymptomatic lipase/amylase elevation), allow ICIs to be maintained for grade 2 pancreatitis (moderate), or suggest prednisone/methylprednisolone (0.5–1 mg/kg/d). For G3–G4 grade pancreatitis (severe and life-threatening), it is recommended to permanently discontinue immunotherapy and start therapy with 1–2 mg/kg/day of glucocorticoids. When the symptoms of ICIs-P improve to grade 1, steroids can be gradually reduced over 4–6 weeks; However, the NCCN guidelines are based on traditional acute pancreatitis rather than ICIs-P evidence; therefore, the current treatment options suggested may not be optimal.

However, the effect of steroids on patients with ICIs-P remains unclear. It has been suggested that there is no clear benefit of steroids in ICIs-P, either in terms of short- or long-term adverse outcomes or improved overall survival (Abu-Sbeih et al., 2019). Notably, Almost all patients in our study had recurrent ICIs-P during steroid reduction. The NCCN guidelines do not provide recommendations for treating steroid-refractory ICIs-P or ICIs-P that recurs during steroid tapering. However, guidelines recommend infliximab for other irAEs if no improvement is observed within 48 h of steroid administration (Thompson et al., 2019). Our report identified two cases of steroid-refractory ICIs-P and four cases of ICIs-P whose prognosis improved after treatment with infliximab and had an inadequate reaction to steroids, suggesting that infliximab may be a potentially feasible therapy for ICIs-P (Cinnor et al., 2017; Townsend et al., 2021). In addition, some authors (Ikeuchi et al., 2016) have stated that patients with severe pancreatitis may require prednisone doses of up to 4 mg/kg/day, arguing that ICIs-P therapy requires not only a high initial dose of systemic glucocorticoids, but also a very slow tapering of the dose. These are the key factors preventing ICIs-P recurrence (Kohlmann et al., 2019).

In addition, after the resolution of irAEs, the decision on whether ICIs should be reinstated should be made cautiously. Guidelines recommend rebooting immunotherapy when harmfulness returns to grade 1 or lower, whereas severe G3–G4 events should discontinue ICIs therapy permanently (Thompson et al., 2019). Some studies have found that survival and tumor remission rates were similar between patients who discontinued ICIs during the introductory phase due to irAEs and those who did not discontinue therapy among patients receiving combination immunotherapy or monotherapy (Schadendorf et al., 2017; Santini et al., 2018). Thus, it is reasonable to administer checkpoint inhibitors treatment to patients with positive reactions but with high-grade irAEs. However, recovery from ICIs treatment is not related to an enhanced risk of long-term adverse consequences with ICIs-P, and patients who resume ICIs therapy have a marginally longer overall survival than those who permanently discontinue ICIs treatment, suggesting that temporary interruption of ICIs followed by resumption after improvement in lipase values may potentially increase the anticancer effect of ICIs (Abu-Sbeih et al., 2019). Our study also found that several patients with G3 grade ICIs-P did not experience pancreatitis recurrence when ICIs was resumed after pancreatitis control (Kohlmann et al., 2019; Goyal et al., 2020; Yamamoto et al., 2021), whereas one patient experienced a new irAE after the resumption of ICIs during treatment (Pagan et al., 2019). Therefore, restarting ICIs therapy after the resolution of high-grade irAEs (e.g., pancreatitis) requires comprehensive consideration according to the condition. Among these, the response status seems to be a critical factor, and reboot immunotherapy should always be performed on a case-by-case basis, specifically in response-deficient or underresponsive patients.

**FIGURE 2:**
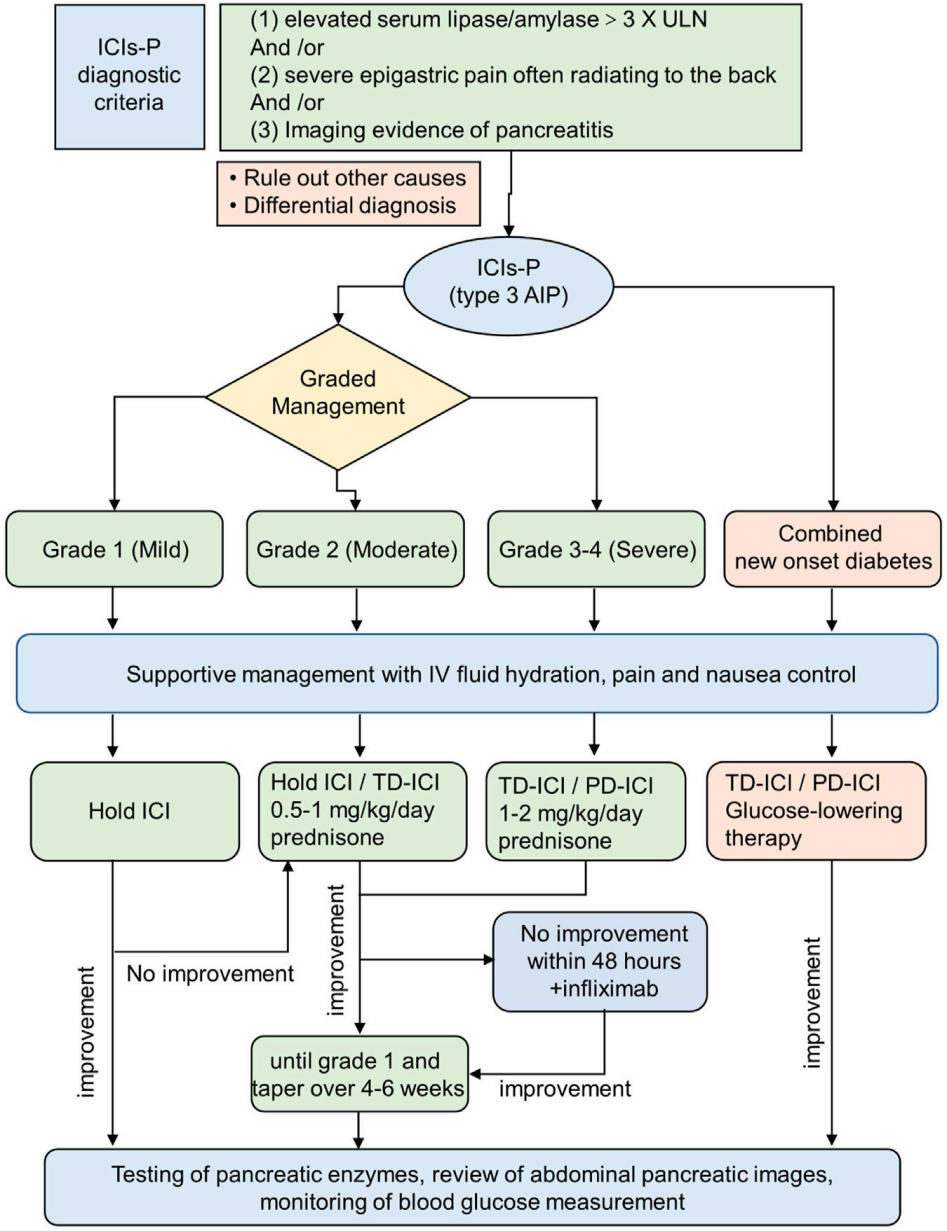
Scheme delineating the approach to the ICIs-P diagnosis and management. The recommended approach reconciles existing guidelines with the newest understanding from clinical experience. ICIs-P,Immune checkpoint inhibitors-induced pancreatitis; TD-ICI,temporary discontinuation of immune checkpoint inhibitors therapy; PD-ICI, permanent discontinuation of immune checkpoint inhibitors therapy.

There are no guidelines for the management of ICIs-P in patients with new-onset diabetes. A review study recommended against the use of glucocorticoids for the treatment of type 1 diabetes associated with ICIs because it may worsen type 1 diabetes and induce ketoacidosis (de Filette et al., 2019). Moreover, patients with acute pancreatitis may become dehydrated due to nausea and vomiting. Therefore, IV fluid is the primary treatment for acute pancreatitis (Waller et al., 2018). The current NCCN guidelines include IV hydration therapy for all ICIs-P grades. Abu-Sbeih et al. noted that aggressive IV rehydration has substantial utility in treating ICIs-P and appears to decrease the risk of long-term adverse events (Abu-Sbeih et al., 2019). We have provided our diagnostic algorithm and therapeutic suggestions based on the available guidelines, published evidence, and our medical practice (Figure 2).

### 4.5 Study limitations

This research has some limitations, mainly because of the retrospective nature of the case reports and FAERS database is a spontaneous reporting system: (1) Risk factors and treatment gaps: Details about risk factors, diagnostic workup, or ICIs-P management may have been missed; this incomplete data limits the identification of potential risk factors and the development of comprehensive strategies. (2) Publication bias: Minor or fatal cases may have been underreported, distorting the understanding of ICIs-P’s true incidence and clinical features, which could misguide clinical decisions and patient counseling. (3) Methodological constraints: The limited sample size, retrospective nature, and prolonged inclusion time introduce heterogeneity in diagnosis and care standards, compromising result reliability, generalizability, and statistical power. (4) Causal inference limitations: The lack of comparator cohorts precludes causal inference and risk stratification in non-pancreatitis populations, hindering clinical interpretation of therapeutic effects and phenotypic correlations. (5) FAERS database limitations: Spontaneously reported adverse events lack proven causality between drugs and events. Incomplete data (e.g., patient history, dosage) further reduce analytical accuracy; FAERS only quantifies reported events, not actual incidence rates. Future studies require multicenter prospective cohorts with standardized data collection to enhance understanding of ICIs-P.

Despite these limitations, to our knowledge, this is the first study integrating FAERS data mining and literature review to assess ICIs-P’s clinical characteristics and risk factors. Additionally, our systematic review includes the largest published cohort of ICI-treated cancer patients with pancreatitis, providing valuable evidence for further research and clinical practice.

## 5 Conclusion

ICIs-P is a rare, but potentially fatal irAEs. Therefore, clinicians should be aware of the risks of pancreatitis associated with immunotherapy and educate patients comprehensively. Early recognition of ICIs-P in the growing number of patients treated with ICIs is critical for its successful management. ICIs-P is an irreversible exocrine autoimmune impairment of the pancreas induced by ICIs that may manifest as asymptomatic, elevated pancreatic enzyme levels, or clinical pancreatitis. Patients with pancreatitis symptoms should have their lipase and amylase levels and radiology evaluated. The diagnosis should be determined by excluding other causes, and the differential diagnosis should consider other types of autoimmune pancreatitis. Steroids are commonly used to treat irAEs associated with checkpoint inhibitors; however, their role in the treatment and prevention of long-term complications of ICIs-P requires further investigation. Rational intravenous rehydration may be beneficial in the treatment of ICIs-P, particularly in combination with new-onset diabetes. Despite some conclusions and recommendations, we recognize the need for further prospective investigations to complement or re-evaluate current treatment policies and expand our understanding of the pathogenesis of pancreatitis associated with checkpoint inhibitors.

## Data Availability

The original contributions presented in the study are included in the article/Supplementary Material, further inquiries can be directed to the corresponding authors.
