# Supplementary material for: Immune checkpoint inhibitors-induced pancreatitis: a systematic review and real-world pharmacovigilance analysis
Source: Front Pharmacol. 2025 Mar 19;16:1426847. doi: 10.3389/fphar.2025.1426847 (PMC11962026; doi:10.3389/fphar.2025.1426847)
Supplement: Supplementary file 1 [file DataSheet2.docx]

***Supplementary Material***

# Immune checkpoint inhibitors-induced pancreatitis: a systematic review and real-world pharmacovigilance analysis

Wei Fang^1†^, Huanping Wang^1†*^, Xiaoran Zhang^2^, Hongxia Zhu^1^, Wei Yan^1^,Yang Gao^3*^

^1^Department of endocrinology, Chengdu Shuangliu Hospital of Traditional Chinese Medicine, Chengdu, China.

^2^Department of Endocrinology, Hospital of Chengdu University of Traditional Chinese Medicine, Chengdu, China.

^3^Laboratory of Ultrasound Medicine, West China Hospital, Sichuan University, Chengdu, China.

*Correspondence:

Yang Gao, gaoyangxueshu@163.com. Huangping Wang,fangw139@163.com.

†These authors contributed equally to this work and share first authorship

Keywords: Immune checkpoint inhibitors, Pancreatitis, Immune-related adverse event, Immunotherapy,pharmacovigilance analysis

## Supplementary Tables.

**Table F1** PTs contained in the narrow-scope search of “Acute pancreatitis (SMQ)”

| PT | MedDRA code |
| --- | --- |
| Cullen's sign | 10059029 |
| Grey Turner's sign | 10075426 |
| Haemorrhagic necrotic pancreatitis | 10076058 |
| Hereditary pancreatitis | 10056976 |
| Idiopathic pancreatitis | 10088882 |
| Immune-mediated pancreatitis | 10083072 |
| Ischaemic pancreatitis | 10066127 |
| Oedematous pancreatitis | 10052400 |
| Pancreatic abscess | 10048984 |
| Pancreatic cyst drainage | 10082531 |
| Pancreatic haemorrhage | 10033625 |
| Pancreatic phlegmon | 10056975 |
| Pancreatic pseudoaneurysm | 10081762 |
| Pancreatic pseudocyst | 10033635 |
| Pancreatic pseudocyst drainage | 10033636 |
| Pancreatic pseudocyst haemorrhage | 10083813 |
| Pancreatic pseudocyst rupture | 10083811 |
| Pancreatitis | 10033645 |
| Pancreatitis acute | 10033647 |
| Pancreatitis haemorrhagic | 10033650 |
| Pancreatitis necrotising | 10033654 |
| Pancreatitis relapsing | 10033657 |
| Pancreatorenal syndrome | 10056277 |
| Subacute pancreatitis | 10084554 |
| Walled-off pancreatic necrosis | 10085347 |

Abbreviations PT, preferred term; MedDRA, Medical Dictionary for Drug Regulatory Activities; SMQ, Standardized MedDRA Querie.

Table F2 Summary of major algorithms used for signal detection

| Algorithms | Equation | Criteria |
| --- | --- | --- |
| ROR | ROR=ad/bc  95%CI=e^ln(ROR)^±1.96(1/a+1/b+1/c+1/d)^0.5 | 95%CI>1, N≥2 |
| PRR | PRR = a(c + d)/c/(a + b)  χ^2^ = [(ad−bc)^2^](a + b + c + d)/[(a + b)(c + d)(a + c)(b + d)] | PRR≥2, χ^2^≥4,  N≥3 |
| BCPNN | IC = log_2_^a(a + b + c + d)/[(a + c)(a + b)]^  IC025=e^ln(IC)-1.96(1/a+1/b+1/c+1/d)^0.5^ | IC025>0 |
| MGPS | EBGM = a(a + b + c + d)/(a + c)/(a + b)  EBGM05=e^ln(EBGM)-1.64(1/a+1/b+1/c+1/d)^0.5^ | EBGM05>2, N>0 |

Abbreviations: a: the number of reports with suspect adverse drug event (ADE) of the suspect drug; b: the number of reports with all other ADEs of the suspect drug; c: the number of reports with the suspect ADE of all other drugs; d: the number of reports with all other ADEs of all other drugs; ROR: reporting odds ratio; CI: confidence interval; N: the number of co-occurrences; PRR: proportional reporting ratio; χ^2^: chi-squared; BCPNN: Bayesian confidence propagation neural network; IC: information component; IC025: the lower limit of the 95% two-sided CI of the IC; MGPS: multi-item gamma Poisson shrinker; EBGM: empirical Bayesian geometric mean; EBGM05: the lower 95% one-sided CI of EBGM

**[Table F3](https://pmc.ncbi.nlm.nih.gov/articles/PMC11600108/#T2)** Clinical characteristics of patients with Immune checkpoint inhibitors-induced pancreatitis collected from the FAERS database

| **Characteristics** | **ICIs/N(%)** | **PD-1/N(%)** | | | | | | | **PD-L1/N(%)** | | | | **CTLA-4/N(%)** | | |
| --- | --- | --- | --- | --- | --- | --- | --- | --- | --- | --- | --- | --- | --- | --- | --- |
|  |  | **Cemiplimab** | **Dostarlimab** | **Nivolumab** | **Pembrolizumab** | **Retifanlimab** | **Toripalimab** | **Total** | **Durvalumab** | **Avelumab** | **Atezolizumab** | **Total** | **Ipilimumab** | **Tremelimumab** | **Total** |
| Gender |  |  |  |  |  |  |  |  |  |  |  |  |  |  |  |
| Male | 307  (50.7%) | 1  (25%) | 0 | 91  (50.8%) | 97  (51.3%) | 0 | 0 | 189  (50.3%) | 15  (55.6%) | 6  (75%) | 40  (37.7%) | 61  (43.3%) | 56  (63.6%) | 1  (100%) | 57  (64%) |
| Female | 217  (35.8%) | 3  (75%) | 1  (50%) | 65  (36.3%) | 83  (43.9%) | 0 | 0 | 152  (40.4%) | 8  (29.6%) | 1  (12.5%) | 28  (26.4%) | 37  (26.2%) | 28  (31.8%) | 0 | 28  (31.5%) |
| Unknown | 82  (13.5%) | 0 | 1  (50%) | 23  (12.8%) | 9  (4.8%) | 0 | 2  (100%) | 35  (9.3%) | 4  (14.8%) | 1  (12.5%) | 38  (35.8%) | 43  (30.5%) | 4  (4.5%) | 0 | 4  (4.5%) |
| Age(years) |  |  |  |  |  |  |  |  |  |  |  |  |  |  |  |
| ≤18 | 2  (0.3%) | 0 | 0 | 0 | 2  (1.1%) | 0 | 0 | 2(0.5%) | 0 | 0 | 0 | 0 | 0 | 0 | 0 |
| 19-45 | 58  (9.6%) | 0 | 0 | 20  (11.2%) | 24  (12.7%) | 0 | 0 | 44  (11.7%) | 2  (7.4%) | 0 | 1  (0.9%) | 3  (2.1%) | 11  (12.5%) | 0 | 11  (12.4%) |
| 46-60 | 106  (17.5%) | 1  (25%) | 0 | 34  (19%) | 38  (20.1%) | 0 | 0 | 73  (19.4%) | 4  (14.8%) | 0 | 10  (9.4%) | 14  (9.9%) | 19  (21.6%) | 0 | 19  (21.3%) |
| 61-80 | 260  (42.9%) | 1  (25%) | 1  (50%) | 75  (41.9%) | 77  (40.7%) | 0 | 0 | 154  (41%) | 13  (48.1%) | 6  (75%) | 38  (35.8%) | 57  (40.4%) | 48  (54.5%) | 1  (100%) | 49  (55.1%) |
| ≥81 | 25  (4.1%) | 1  (25%) | 0 | 2  (1.1%) | 9  (4.8%) | 0 | 0 | 12  (3.2%) | 0 | 1  (12.5%) | 9  (8.5%) | 10  (7.1%) | 3  (3.4%) | 0 | 3  (3.4%) |
| Unknown | 155  (25.6%) | 1  (25%) | 1  (50%) | 48  (26.8%) | 39  (20.6%) | 0 | 2  (100%) | 91  (24.2%) | 8  (29.6%) | 1  (12.5%) | 48  (45.3%) | 57  (40.4%) | 7  (8%) | 0 | 7  (7.9%) |
| Reported  countries |  |  |  |  |  |  |  |  |  |  |  |  |  |  |  |
| UNITED STATES | 186  (30.7%) | 1  (25%) | 1  (50%) | 64  (35.8%) | 68  (36%) | 0 | 0 | 134  (35.6%) | 5  (18.5%) | 3  (37.5%) | 21  (19.8%) | 29  (20.6%) | 23  (26.1%) | 0 | 23  (25.8%) |
| JAPAN | 236  (38.9%) | 1  (25%) | 0 | 51  (28.5%) | 74  (39.2%) | 0 | 0 | 126  (33.5%) | 10  (37%) | 3  (37.5%) | 50  (47.2%) | 63  (44.7%) | 46  (52.3%) | 1  (100%) | 47  (52.8%) |
| FRANCE | 40  (6.6%) | 0 | 0 | 14  (7.8%) | 13  (6.9%) | 0 | 0 | 27  (7.2%) | 4  (14.8%) | 0 | 5  (4.7%) | 9  (6.4%) | 4  (4.5%) | 0 | 4  (4.5%) |
| GERMANY | 36  (5.9%) | 0 | 0 | 18  (10.1%) | 9  (4.8%) | 0 | 0 | 27  (7.2%) | 0 | 1  (12.5%) | 5  (4.7%) | 6  (4.3%) | 3  (3.4%) | 0 | 3  (3.4%) |
| AUSTRALIA | 14  (2.3%) | 0 | 0 | 4  (2.2%) | 3  (1.6%) | 0 | 0 | 7  (1.9%) | 0 | 0 | 2  (1.9%) | 2  (1.4%) | 5  (5.7%) | 0 | 5  (5.6%) |
| UNITED KINGDOM | 2  (0.3%) | 0 | 0 | 2  (1.1%) | 0 | 0 | 0 | 2  (0.5%) | 0 | 0 | 0 | 0 | 0 | 0 | 0 |
| Other Countries | 0 | 2  (50%) | 1  (50%) | 26  (14.5%) | 22  (11.6%) | 0 | 2  (100%) | 53  (14.1%) | 8  (29.6%) | 1  (12.5%) | 23  (21.7%) | 32  (22.7%) | 7  (8%) | 0 | 7  (8%) |
| Outcome |  |  |  |  |  |  |  |  |  |  |  |  |  |  |  |
| Death | 88  (14.5%) | 0 | 0 | 24  (13.4%) | 29  (15.3%) | 0 | 1  (50%) | 54  (14.4%) | 5  (18.5%) | 2  (25%) | 17  (16%) | 24  (17%) | 10  (11.4%) | 0 | 10  (11.2%) |
| Hospitalization | 231  (38.1%) | 0 | 2  (100%) | 61  (34.1%) | 77  (40.7%) | 0 | 1  (50%) | 141  (37.5%) | 8  (29.6%) | 4  (50%) | 31  (29.2%) | 43  (30.5%) | 47  (53.4%) | 0 | 47  (52.8%) |
| Disability | 2  (0.3%) | 0 | 0 | 0 | 1  (0.5%) | 0 | 0 | 1  (0.3%) | 0 | 0 | 0 | 0 | 1  (1.1%) | 0 | 1  (1.1%) |
| Life-Threatening | 46  (7.59%) | 2  (50%) | 0 | 21  (11.73%) | 8  (4.23%) | 0 | 0 | 31  (8.24%) | 4  (14.81%) | 0 | 3  (2.83%) | 7  (4.96%) | 7  (7.95%) | 1(100%) | 8  (8.99%) |
| Other | 239  (39.44%) | 2  (50%) | 0 | 73  (40.78%) | 74  (39.15%) | 0 | 0 | 149  (39.63%) | 10  (37.04%) | 2  (25%) | 55  (51.89%) | 67  (47.52%) | 23  (26.14%) | 0 | 23  (25.84%) |

**Abbreviations:** N,number; ICIs,Immune checkpoint inhibitors; PD-1, programmed cell death protein 1; PD-L1, programmed death-ligand 1; CTLA-4, cytotoxic T lymphocyte antigen 4.


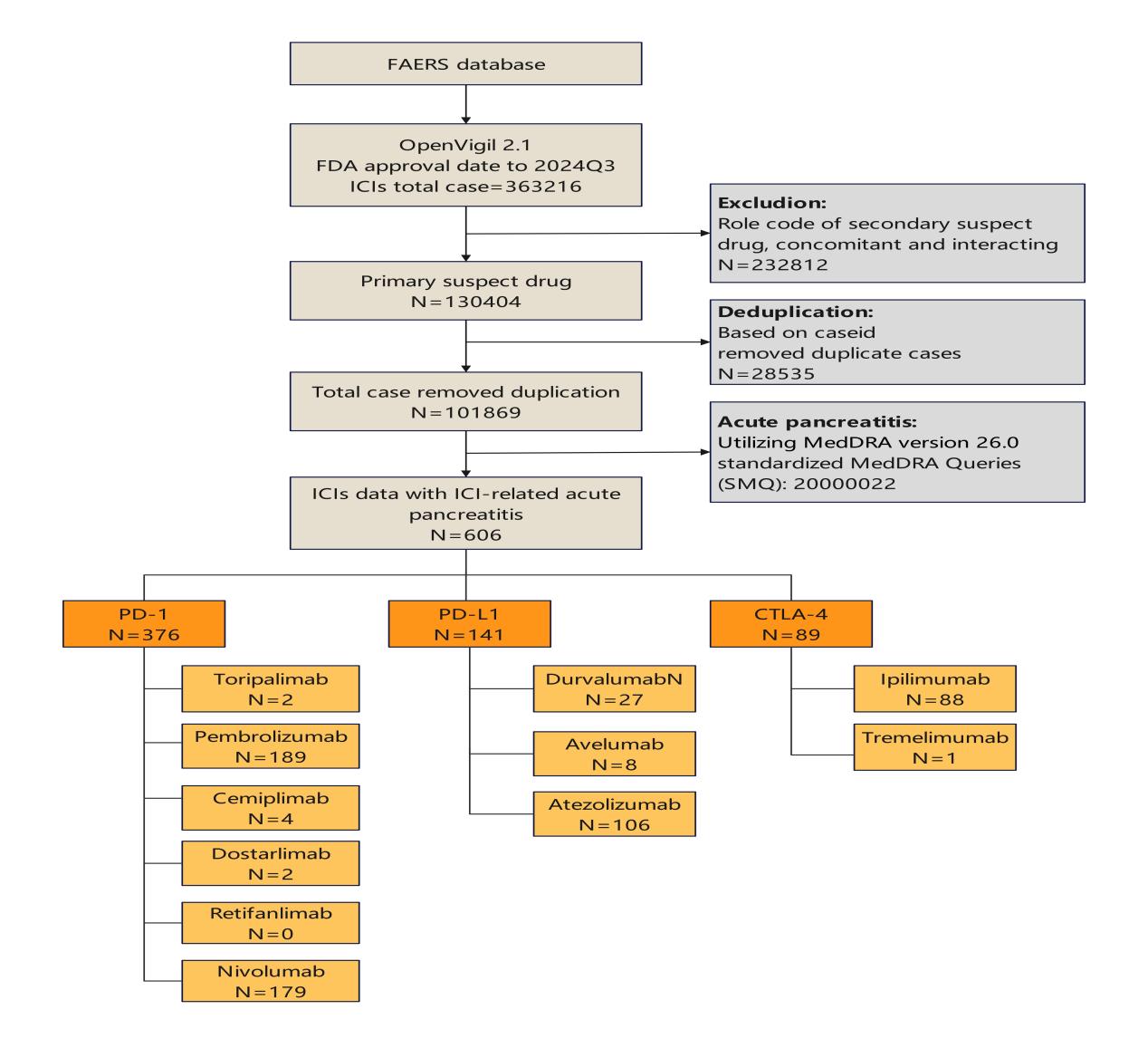


**Fig. F1.** The data filtering procedure employed in this study
