# Supplementary material for: Immune checkpoint inhibitors-induced pancreatitis: a systematic review and real-world pharmacovigilance analysis
Source: Front Pharmacol. 2025 Mar 19;16:1426847. doi: 10.3389/fphar.2025.1426847 (PMC11962026; doi:10.3389/fphar.2025.1426847)
Supplement: Supplementary file 2 [file DataSheet1.docx]

***Supplementary Material***

# Immune checkpoint inhibitors-induced pancreatitis: a systematic review and real-world pharmacovigilance analysis

Wei Fang^1†^, Huanping Wang^1†*^, Xiaoran Zhang^2^, Hongxia Zhu^1^, Wei Yan^1^,Yang Gao^3*^

^1^Department of endocrinology, Chengdu Shuangliu Hospital of Traditional Chinese Medicine, Chengdu, China.

^2^Department of Endocrinology, Hospital of Chengdu University of Traditional Chinese Medicine, Chengdu, China.

^3^Laboratory of Ultrasound Medicine, West China Hospital, Sichuan University, Chengdu, China.

*Correspondence:

Yang Gao, gaoyangxueshu@163.com. Huangping Wang,fangw139@163.com.

†These authors contributed equally to this work and share first authorship

Keywords:Immune checkpoint inhibitors, Pancreatitis, Immune-related adverse event, Immunotherapy, pharmacovigilance analysis

## Supplementary Tables.

**Table S1.**Search strategy in four databases

| Database | Search Details | Results |
| --- | --- | --- |
| **PubMed** | #1:((((((((((((((((((((((((((((((((((((((((((((((((((((((pembrolizumab[Title/Abstract]) OR (lambrolizumab[Title/Abstract])) OR (Keytruda[Title/Abstract])) OR (MK-3475[Title/Abstract])) OR (nivolumab[Title/Abstract])) OR (MDX-1106[Title/Abstract])) OR (ONO-4538[Title/Abstract])) OR (BMS-936558[Title/Abstract])) OR (Opdivo[Title/Abstract])) OR (atezolizumab[Title/Abstract])) OR (MPDL3280A[Title/Abstract])) OR (Tecentriq[Title/Abstract])) OR (RG7446[Title/Abstract])) OR (RG 7446[Title/Abstract])) OR (camrelizumab[Title/Abstract])) OR (SHR 1210[Title/Abstract])) OR (SHR-1210[Title/Abstract])) OR (durvalumab[Title/Abstract])) OR (MEDI4736[Title/Abstract])) OR (MEDI-4736[Title/Abstract])) OR (Imfinzi[Title/Abstract])) OR (toripalimab[Title/Abstract])) OR (sintilimab[Title/Abstract])) OR (IBI 308[Title/Abstract])) OR (IBI308[Title/Abstract])) OR (IBI-308[Title/Abstract])) OR (tislelizumab[Title/Abstract])) OR (ipilimumab[Title/Abstract])) OR (Yervoy[Title/Abstract])) OR (MDX 010[Title/Abstract])) OR (MDX010[Title/Abstract])) OR (MDX-010[Title/Abstract])) OR (MDX-CTLA-4[Title/Abstract])) OR (MDX CTLA 4[Title/Abstract])) OR (immune checkpoint inhibitors[Title/Abstract])) OR (ICIs[Title/Abstract])) OR (immune checkpoint blockade[Title/Abstract])) OR (ICB[Title/Abstract])) OR (anti-PD1[Title/Abstract])) OR (PD-1[Title/Abstract])) OR (Programmed Death 1[Title/Abstract])) OR (Programmed Cell Death 1 Receptor[Title/Abstract])) OR (PD 1[Title/Abstract])) OR (PD1[Title/Abstract])) OR (Programmed Death-Ligand 1[Title/Abstract])) OR (PD-L1[Title/Abstract])) OR (programmed cell death 1 ligand 1 protein[Title/Abstract])) OR (PD L1[Title/Abstract])) OR (PDL1[Title/Abstract])) OR (CTLA-4[Title/Abstract])) OR (CD152[Title/Abstract])) OR (CTLA-4 Protein[Title/Abstract])) OR (CTLA 4 Protein[Title/Abstract])) OR (Cytotoxic T-Lymphocyte Antigen 4[Title/Abstract])) OR (Cytotoxic T Lymphocyte Antigen 4[Title/Abstract]) | 96379 |
|  | #2:((((((Pancreatitis[MeSH Terms]) OR (Pancreatitis, Acute Necrotizing[Title/Abstract])) OR (Pancreatitis, Acute Hemorrhagic[Title/Abstract])) OR ((acute adj2 pancrea*[Title/Abstract]))) OR ((necro* adj2 pancrea*[Title/Abstract]))) OR ((inflam* adj3 pancrea*[Title/Abstract]))) OR (((interstitial[Title/Abstract] OR edema*[Title/Abstract]) adj2 pancrea*)) | 58639 |
|  | #3:#1 AND #2 | 100 |
| **embase** | #1:'pembrolizumab'/exp OR 'pembrolizumab' OR 'pembrolizumab'/exp OR pembrolizumab OR lambrolizumab:ab,ti OR keytruda:ab,ti OR 'mk 3475':ab,ti OR nivolumab:ab,ti OR 'mdx 1106':ab,ti OR 'ono 4538':ab,ti OR 'bms 936558':ab,ti OR opdivo:ab,ti OR atezolizumab:ab,ti OR mpdl3280a:ab,ti OR tecentriq:ab,ti OR rg7446:ab,ti OR 'rg 7446':ab,ti OR camrelizumab:ab,ti OR 'shr 1210':ab,ti OR durvalumab:ab,ti OR medi4736:ab,ti OR 'medi 4736':ab,ti OR imfinzi:ab,ti | 74,047 |
|  | #2:'toripalimab'/exp OR toripalimab OR sintilimab:ab,ti OR ibi308:ab,ti OR 'ibi 308':ab,ti OR tislelizumab:ab,ti OR ipilimumab:ab,ti OR mdx010:ab,ti OR 'mdx 010':ab,ti OR 'mdx ctla 4':ab,ti OR 'immune checkpoint inhibitors':ab,ti OR ICIs:ab,ti OR icb:ab,ti OR 'anti pd1':ab,ti OR 'programmed death 1':ab,ti OR 'programmed cell death 1 receptor':ab,ti OR 'pd 1':ab,ti OR pd1:ab,ti OR 'programmed death-ligand 1':ab,ti OR 'programmed cell death 1 ligand 1 protein':ab,ti OR 'pd l1':ab,ti OR pdl1:ab,ti OR 'ctla 4':ab,ti OR cd152:ab,ti OR 'ctla-4 protein':ab,ti OR 'ctla 4 protein':ab,ti OR 'cytotoxic t-lymphocyte antigen 4':ab,ti | 152809 |
|  | #3:#1 OR #2 | 183358 |
|  | #4:'pancreatitis'/exp OR pancreatitis OR 'pancreatitis, acute necrotizing':ab,ti OR 'pancreatitis, acute hemorrhagic':ab,ti OR 'acute adj2 pancrea*':ab,ti OR 'necro* adj2 pancrea*':ab,ti OR 'inflam* adj3 pancrea*':ab,ti OR ((interstitial:ab,ti OR edema*:ab,ti) AND 'adj2 pancrea*':ab,ti) | 129721 |
|  | #5:#3 AND #4 | 1389 |
| **Cochrane** | #1:(pembrolizumab):ti,ab,kw OR (lambrolizumab):ti,ab,kw OR (Keytruda):ti,ab,kw OR (MK-3475):ti,ab,kw OR (nivolumab):ti,ab,kw (Word variations have been searched) OR (MDX-1106):ti,ab,kw OR (ONO-4538):ti,ab,kw OR (BMS-936558):ti,ab,kw OR (Opdivo):ti,ab,kw OR (atezolizumab):ti,ab,kw (Word variations have been searched) OR (MPDL3280A):ti,ab,kw OR (Tecentriq):ti,ab,kw OR (RG7446):ti,ab,kw OR (RG 7446):ti,ab,kw OR (camrelizumab):ti,ab,kw (Word variations have been searched) OR (SHR-1210):ti,ab,kw OR (SHR 1210):ti,ab,kw OR (durvalumab):ti,ab,kw OR (MEDI4736):ti,ab,kw OR (MEDI-4736):ti,ab,kw (Word variations have been searched) OR (Imfinzi):ti,ab,kw OR (toripalimab):ti,ab,kw OR (sintilimab):ti,ab,kw OR (IBI 308):ti,ab,kw OR (IBI308):ti,ab,kw (Word variations have been searched) OR (IBI-308):ti,ab,kw OR (tislelizumab):ti,ab,kw OR (ipilimumab):ti,ab,kw OR (Yervoy):ti,ab,kw OR (MDX 010):ti,ab,kw OR  (MDX010):ti,ab,kw OR (MDX-010):ti,ab,kw OR (MDX-CTLA-4):ti,ab,kw OR (MDX CTLA 4):ti,ab,kw OR (immune checkpoint inhibitors):ti,ab,kw OR (ICIs):ti,ab,kw OR (immune checkpoint blockade):ti,ab,kw OR (ICB):ti,ab,kw OR (anti-PD1):ti,ab,kw AND (PD-1):ti,ab,kw OR (Programmed Death 1):ti,ab,kw OR (Programmed Cell Death 1 Receptor):ti,ab,kw OR (PD 1):ti,ab,kw OR (PD1):ti,ab,kw OR (Programmed Death-Ligand 1):ti,ab,kw OR (PD-L1):ti,ab,kw OR (programmed cell death 1 ligand 1 protein):ti,ab,kw OR (PD L1):ti,ab,kw OR (PDL1):ti,ab,kw OR (CTLA-4):ti,ab,kw OR (CD152):ti,ab,kw OR (CTLA-4):ti,ab,kw OR (CTLA 4):ti,ab,kw OR (Cytotoxic T-Lymphocyte Antigen 4):ti,ab,kw OR (Cytotoxic T Lymphocyte Antigen 4):ti,ab,kw (Word variations have been searched) | 39674 |
|  | #2:(Pancreatitis):ti,ab,kw OR (Pancreatitis, Acute Necrotizing):ti,ab,kw OR (Pancreatitis, Acute Hemorrhagic):ti,ab,kw OR ((acute adj2 pancrea*)):ti,ab,kw OR ((necro* adj2 pancrea*)):ti,ab,kw (Word variations have been searched) OR ((inflam* adj3 pancrea*)):ti,ab,kw OR (((interstitial or edema*) adj2 pancrea*)):ti,ab,kw (Word variations have been searched) | 22774 |
|  | #3:#1 AND #2 | 988 |
| **Web of**  **Science** | #1:(((((((((((((((((((((((((((((((((((((((((((((((((((AB=(pembrolizumab)) OR AB=(lambrolizumab)) OR AB=(Keytruda)) OR AB=(MK-3475)) OR AB=(nivolumab)) OR AB=(MDX-1106)) OR AB=(ONO-4538)) OR AB=(BMS-936558)) OR AB=(Opdivo)) OR AB=(atezolizumab)) OR AB=(MPDL3280A)) OR AB=(Tecentriq)) OR AB=(RG7446)) OR AB=(RG 7446)) OR AB=(camrelizumab)) OR AB=(SHR 1210)) OR AB=(SHR-1210)) OR AB=(durvalumab)) OR AB=(MEDI4736)) OR AB=(MEDI-4736)) OR AB=(Imfinzi)) OR AB=(toripalimab)) OR AB=(sintilimab)) OR AB=(IBI 308)) OR AB=(IBI308)) OR AB=(IBI-308)) OR AB=(tislelizumab)) OR AB=(ipilimumab)) OR AB=(MDX 010)) OR AB=(MDX010)) OR AB=(MDX-010)) OR AB=(MDX-CTLA-4)) OR AB=(MDX CTLA 4)) OR AB=(immune checkpoint inhibitors)) OR AB=(ICIs)) OR AB=(immune checkpoint blockade)) OR AB=(ICB)) OR AB=(anti-PD1)) OR AB=(PD-1)) OR AB=(Programmed Death 1)) OR AB=(Programmed Cell Death 1 Receptor)) OR AB=(PD 1)) OR AB=(PD1)) OR AB=(Programmed Death-Ligand 1)) OR AB=(PD-L1)) OR AB=(programmed cell death 1 ligand 1 protein)) OR AB=(PDL1)) OR AB=(CTLA-4)) OR AB=(CD152)) OR AB=(CTLA-4 Protein)) OR AB=(CTLA 4 Protein)) OR AB=(Cytotoxic T-Lymphocyte Antigen 4) | 346780 |
|  | #2: ((((((AB=(Pancreatitis, Acute Necrotizing)) OR AB=(Pancreatitis)) OR AB=(Pancreatitis, Acute Hemorrhagic)) OR AB=((acute adj2 pancrea*))) OR AB=((necro* adj2 pancrea*))) OR AB=((inflam* adj3 pancrea*))) OR AB=(((interstitial or edema*) adj2 pancrea*)) | 77033 |
|  | #3: #2 AND #1 | 850 |

**Table S2.**Published case reports and case series of immune checkpoint inhibitors associated with pancreatitis

| **Study**  **(year)** | **Diagnosis** | **Age** | **Sex** | **Agent（ICIs)** | **Time to onset of pancreatitis after ICIs** | **Past medical history** | **Symptoms** | **IgG4**  (mg/dL) | **Lipase**  **(U/L)** | **Amylases (U/L)** | **Grade** | **Pancreatic imaging** | **Treatment of pancreatitis** | **Discontinued ICIs** | **Other irAEs** | **Outcome** |
| --- | --- | --- | --- | --- | --- | --- | --- | --- | --- | --- | --- | --- | --- | --- | --- | --- |
| Satoshi Tanaka  2020[1] | NSCLC  (IVA) | 65 | M | Pembrolizumab | 2 cycles  （3 weeks/ cycles） | Smoker  (30 pack- years) | abdominal pain | NR | 167 | 153 | G3 | diffuse pancreatic enlargement（CT） | fasting and intravenous fluids. | No  (PD) | hepatitis | improved |
| Evelien van Gogh  2018[2] | uveal melanoma | 53 | M | Nivolumab/  Ipilimumab^*^ | 6 cycles  （2 weeks/ cycles）/12 day | None | acute epigastric pain and nausea | NR | 7270 | NR | G2 | no signs of pancreatitis  (CAT) | MLL（80 mg IV bid）[only]/ infliximab  (5m/kg) | Hold | None | improved |
| Chandler, N  2021[3] | IDC  (III) | 54 | F | Atezolizumab | 3 cycles | None | asymptomatic | NR | 1109 | 293 | G2 | minimal peripancreatic inflammatory changes（CT） | prednisone  (1 mg/kg/day)  [only] | Yes  (TDR) | None | improved |
| Koichiro Yamamoto  2021[4] | melanoma | 76 | M | Nivolumab +Ipilimumab^#^ | 2 cycles,  8 weeks  （3 weeks/ cycles） | hypertensio, dyslipidemi,  and hyperuricaemia | general fatigue, anorexia, and diarrhoea. | Normal  (69.2) | 1520 | 683 | G3 | enlarged pancreatic parenchyma(CECT)/ peripancreatic fat stranding (MRCP) | PLS 30mg  (0.5 mg/kg/day)  [only] | Yes  (TDR) | moderate dermatitis | improved |
| Takeshi Tanaka  2021[5] | RCC | 74 | F | Nivolumab | 1 year | Right nephrectomy | abdominal pain | Normal  (28) | 8788 | 4050 | G4 | diffuse pancreatic swelling(CE-CT) | conservative treatment+MLL 40mg (1 mg/kg) [only] | N/A | None | Death※1 |
| Jiang, R  2018[6] | squamous carcinoma  (IIA) | 64 | M | Nivolumab | 1 day | None | abdominal pain | NR | NR | 253 | G4 | exudative lesions(CT) | comprehensive therapy | N/A | None | Death※1 |
| Preeya Goyal  2020[7] | metastatic acral melanoma | 60 | M | Nivolumab+  Ipilimumab | 4 cycles  （3 weeks/ cycles） | None | mild abdominal discomfort | Normal | 517 | NR | G3 | diffuse mild enlargement of the pancreas(CT) | intravenous fluids+MLL  (0.5mg/kg/day）[only] | Yes  (TDR) | None | Recurrence |
| Masayuki Ueno  2021[8] | NSCLC | 62 | M | Pembrolizumab | At three months after 12 cycles | asthma, chronic obstructive pulmonary disease, and depression | epigastric pain | 133 (11-121) | 105 | 179 | G3 | Enlarged pancreas(CE-CT) | fluid therapy+  Corticosteroids  [also] | No  (PD) | Hypothyroidism, pneumonitis | Death※2 |

**Table S2**.(continued)

| **Study**  **(year)** | **Diagnosis** | **Age** | **Sex** | **Agent（ICIs)** | **Time to onset of pancreatitis after ICIs** | **Past medical history** | **Symptoms** | **IgG4**  (mg/dL) | **Lipase**  **(U/L)** | **Amylases (U/L)** | **Grade** | **Pancreatic imaging** | **Treatment of pancreatitis** | **Discontinued ICIs** | **Other irAEs** | **Outcome** |
| --- | --- | --- | --- | --- | --- | --- | --- | --- | --- | --- | --- | --- | --- | --- | --- | --- |
| Birtukan cinnor  2017[9] | metastatic melanoma | 60 | F | Ipilimumab/ Pembrolizumab | 9 cycles | None | abdominal pain, nausea, and intractable vomiting | NR | 656 | NR | G3 | mild pancreatitis(CT) | MLL[also]+  Infliximab  (5mg/kg) | No  (PD) | Hemorrhag-ic gastritis | improved |
| Gabriele Capurso  2018[10] | urothelial carcinoma | 76 | F | Pembrolizumab | NR | R0 nephrectomy | diarrhoea and weight loss | Normal | Normal | Normal | G2 | Stricture and upstream dilation of MPD+a diffuse signal restriction(CT) | pancreatic enzyme replacement therapy (PERT) | TAC | None | improved |
| Jeeban Paul Das  2019[11] | lung adenocarcinoma | 46 | M | Pembrolizumab | 3 cycles | None | epigastric pain | NR | Elevation | Elevation | G3 | pancreatic tail enlargement(CT) | Corticosteroids  [only] | No  (PD) | None | improved |
| Pagan, Andrea  2019[12] | Stage IV oligometastatic clear cell renal carcinoma | 76 | M | Pembrolizumab | NR | hypertension and COPD | abdominal pain, nausea, and vomiting | NR | 436 | NR | G3 | edematous pancreas with loss of pancreatic lobulation(CT) | Steroid  [only] | Hold | colitis | improved |
| Preeya Goyal  2020[13] | melanoma metastatic | 60 | M | Nivolumab + Ipilimumab | 4 cycles | None | abdominal discomfort | Normal | 517 | NR | G3 | mild diffuse enlargement of the pancreas  (CT) | intravenous fluids + corticosteroids  [only] | NR | cholestatic liver disease | Recurrence |
| Johannes Kohlmann  2019[14] | melanoma | 58 | M | Ipilimumab+  Nivolumab | 4 cycles(106 days) | None | a belt-shaped epigastric pain | NR | 394.2 | 318 | G3 | edematous swelling both within the pancreatic tail(CT) | MLL  (1.3mg/kg)  [only] | Yes  (TDR) | oral mucositis and intermittent diarrhea | Recurrence |
| Zhang, H C  2019[15] | metastatic melanoma | 23 | M | Ipilimumab + Nivolumab | 10 weeks | None | epigastric pain | Normal | 4418 | 465 | G3 | heterogeneous pancreas surrounded by minimal fluid  (MRI/MRCP) | pancrelipase | No  (PD) | colitis | improved |
| Kazuhiko Ikeuchi  2016[16] | esophageal squamous cell carcinoma and lung adenocarcinoma | 66 | F | Nivolumab | 2 cycles  (18 days) | None | anorexia, vomiting, and back pain | NR | Elevation | Elevation | G3 | no pancreatic abnormalities  (CT and MRCP) | Intravenous fluid hydration  +ulinastatin+  Prednisone  (4 mg/kg/day)  [also] | NR | hepatitis, and nephritis | improved |
| Townsend, Matthew J.  2021[17] | melanoma metastatic to the lung | 63 | F | Pembrolizumab | 15 cycles | Hyperlipase-mia | asymptomatic | NR | Elevation | elevation | G2 | diffuse pancreatic enlargement and fat stranding(CT) | MLL(1mg/kg）/Infliximab(5mg/kg) | NR | None | Recurrence |

**Table S2**.(continued)

| **Study**  **(year)** | **Diagnosis** | **Age** | **Sex** | **Agent（ICIs)** | **Time to onset of pancreatitis after ICIs** | **Past medical history** | **Symptoms** | **IgG4**  (mg/dL) | **Lipase**  **(U/L)** | **Amylases (U/L)** | **Grade** | **Pancreatic imaging** | **Treatment of pancreatitis** | **Discontinued ICIs** | **Other irAEs** | **Outcome** |
| --- | --- | --- | --- | --- | --- | --- | --- | --- | --- | --- | --- | --- | --- | --- | --- | --- |
| Somasundaram, A  2015[18] | stage III melanoma of scalp | 45 | F | Ipilimumab + Pembrolizumab | 4 cycles/  5 cycles | Hyperlipase-mia | abdominal pain, intractable nausea, vomiting, and watery diarrhea | NR | 1880 | NR | G3 | Pancreatitis  ( PET-CT) | intravenous fluids+MLL  [also] | TAC | thyroiditis | improved |
| Christine Newman  2021[19] | malignant melanoma | 46 | F | Nivolumab + Ipilimumab | 8 cycles  (30 weeks) | None | asymptomatic | NR | Normal | Normal | G1 | acute pancreatitis(CT) | intravenous hydration+  PLS  [also] | Yes  (TDR) | Hypophysi-tis, thyroiditis, hypercalca-emia | improved |
| Morihisa Hirota  2022[20] | squamous cell carcinoma of the lung | 56 | M | Pembrolizumab | 13 cycles | Smoker  (20/day  - 35 years) | upper abdominal pain | Normal  (54.0) | 2273 | 556 | G4 | diffuse enlargement of the pancreas with peripancreatic fat stranding  (CE-CT） | Steroid(1 mg/kg/day)  [also] | No  (PD) | Colitis | improved |
| Wesley Rogers  2019[21] | small cell lung cancer | 61 | M | Nivolumab | 40 days | None | epigastric pain radiating | NR | 2700 | NR | G4 | acute interstitial pancreatitis（CT） | Hydromorphon-e,  Lactated Ringer’s and  Dexamethasone  [only] | NR | None | Death※3 |
| Hiroaki Saito  2019[22] | metastatic squamous cell carcinoma of the lung | 72 | M | Nivolumab | NR | None | asymptomatic | NR | Elevation | Elevation | G2 | Enlarged pancreas  (PET/CT) | cessation of nivolumab therapy | No  (PD) | None | improved |
| Tanaka, T.  2019[23] | stage IV renal cell carcinoma | 70 | F | Nivolumab | 6 months | None | NR | NR | 1830 | 547 | G2 | diffuse enlargement of the whole pancreas（MRI） | Discontinuation of nivolumab therapy | No  (PD) | None | improved |
| Laurens Janssens  2021[24] | metastatic malignant melanoma | 38 | F | Nivolumab | 4 months | None | epigastric pain | normal | 439 | 146 | G3 | heterogeneous parenchymal enhancement in the pancreatic body（CT） | Discontinuation of nivolumab+  Prednisone （40mg/day）[only] | No  (PD) | None | improved |

**Table S2**.(continued)

| **Study**  **(year)** | **Diagnosis** | **Age** | **Sex** | **Agent（ICIs)** | **Time to onset of pancreatitis after ICIs** | **Past medical history** | **Symptoms** | **IgG4**  (mg/dL) | **Lipase**  **(U/L)** | **Amylases (U/L)** | **Grade** | **Pancreatic imaging** | **Treatment of pancreatitis** | **Discontinued ICIs** | **Other irAEs** | **Outcome** |
| --- | --- | --- | --- | --- | --- | --- | --- | --- | --- | --- | --- | --- | --- | --- | --- | --- |
| M Domínguez Bachiller  2020[25] | non-small cell lung cancer | 67 | M | Nivolumab | 18 months | None | abdominal pain | NR | Elevation | Elevation | G3 | Pancreatitis  （Gastroscopy） | Cortico therapy  [only] | NR | None | improved |
| M Domínguez Bachiller  2020[25] | carcinoma of probable pulmonary origin | 58 | F | Pembrolizumab | 25 cycles | None | abdominal pain and vomiting | NR | NR | NR | G2 | Pancreatitis  （CAT） | fluid therapy+steroid therapy  [only] | NR | None | improved |
| Yazan Z. Alabed  2015[26] | metastatic melanoma | 57 | M | Ipilimumab/  Pembrolizumab | 3 cycles/  3 cycles | None | Asymptomatic | NR | 205 | 107 | G2 | Fat stranding around the pancreas  （CT） | corticosteroid therapy[only] | NR | colitis | improved |
| Kazuya Ofuji  2021[27] | large cell lung cancer | 82 | M | Pembrolizumab | 6 cycles | smoking and alcohol consumption | fever | Normal  (54) | 1007 | NR | G3 | diffuse enlargement of the entire pancreas(CT) | PSL  (1.0 mg/kg/day)  [also] | NR | UC-like colitis | improved |
| Victor Delgado-Lazo  2022[28] | colon cancer  (IV) | 43 | M | Pembrolizumab | 7 months | type 2 diabetes | abdominal pain | NR | 588 | NR | G3 | peripancreatic stranding and haziness(CT) | analgesia hydration and prednisone  [also] | No  (PD) | Myocarditis | improved |
| Tamaki Kakuwa  2020[29] | lung squamous cell carcinoma | 70 | M | Pembrolizumab | 14 months | None | Asymptomatic | NR | Elevation | Elevation | G2 | a slightly swollen pancreatic parenchyma and mild pancreatic duct dilation(CT) | PSL  (1 mg/kg/day)  [only] | NR | None | improved |
| Robert V. Rawson  2019[30] | metastatic melanoma | 43 | M | Ipilimumab/  Pembrolizumab | 3 cycles  (3 weeks  /cycles)/  11 cycles  (3 weeks  /cycles) | NR | NR | Normal | Normal  （55） | NR | G1 | no pancreatic mass identified  (MRI） | Dexamethasone  [also] | Yes  (TDR) | small bowel obstructions | improved |
| H. Kim  2020[31] | melanoma | 43 | F | Pembrolizumab | 9 cycles | None | anorexia, nausea, vomiting, and severe epigastric pain | NR | 485.0 | 313.5 | G3 | diffuse gastroduodenitis with acute pancreatitis  (PET/CT) | steroid therapy  (dexamethasone 10 mg)  [only] | NR | cytomegalovirus gastritis | improved |
| Tsuyoshi Suda  2021[32] | lung cancer | 57 | M | Pembrolizumab | 6 weeks | None | epigastric pain | Normal | NR | 332 | G3 | pancreatic swelling  (CE-CT) | MLL  (120 mg/day) [also]and nafamostat mesylate | NR | liver injury | improved |

**Table S2**.(continued)

| **Study**  **(year)** | **Diagnosis** | **Age** | **Sex** | **Agent（ICIs)** | **Time to onset of pancreatitis after ICIs** | **Past medical history** | **Symptoms** | **IgG4**  (mg/dL) | **Lipase**  **(U/L)** | **Amylases (U/L)** | **Grade** | **Pancreatic imaging** | **Treatment of pancreatitis** | **Discontinued ICIs** | **Other irAEs** | **Outcome** |
| --- | --- | --- | --- | --- | --- | --- | --- | --- | --- | --- | --- | --- | --- | --- | --- | --- |
| Tomotaka Yazaki  2022[33] | left renal cell carcinoma | 62 | M | Nivolumab | 9 cycles | Left nephrectomy | fever, nausea, and abdominal fullness | Normal | 248 | 200 | G3 | not show positive findings  (CT) | fasting + a proteolytic enzyme inhibitor+  PSL  (1 mg/kg/day)  [also] | No  (PD) | colitis | improved |
| Anna Maria Di Giacomo  2009[34] | Cutaneous melanoma | 36 | F | Ipilimumab | 7 weeks | gallstones-  related pancreatitis | abdominal pain | NR | Elevation | Elevation | G3 | acute pancreatitis  (MRCP) | Dexamethasone(8mg)/prednisone(25mg)[also] | No  (PD) | colitis | improved |
| Rogers  2020[35] | squamous cell lung cancer  (IIIB) | 55 | M | Duvalumab | 15 cycles | hypertension, diabetes, and hyperlipide-mia | upper abdominal discomfort,  nausea | NR | Elevation | Elevation | G2 | NR | No treatment | Hold | None | improved |
| Lea Dehghani  2018[36] | metastatic melanoma | 63 | M | Nivolumab | 15 months | None | asymptomatic | 0.33  (0.04–0.86) | 63 | NR | G2 | peripancreatic fatty infiltration (CT) | Insulin and Pancreatic enzyme replacement therapy | TAC | Liver injury | improved |
| Mesut Yilmaz  2021[37] | mRCC(IIIc) | 49 | M | Nivolumab | 34 months  (72 cycles) | left radical nephrectomy and retroperitoneal lymph node dissection | nausea, vomiting, and abdominal pain | NR | 1587 | 728 | G3 | diffuse enlargement of the whole pancreas (CT) | Methylprednisolone  (iv, 2mg/kg) [only] | No  (PD) | None | improved |
| Wataru Munakata  2016[38] | cHL | 72 | M | Nivolumab | 6 cycles | None | slight thirst, polyuria, and general fatigue | NR | 80 | NR | G1 | diffusely enlarged (MRI) | intensive insulin replacement therapy | Yes  (TDR) | None | improved |
| [Kiyokuni Tanabe](https://pubmed.ncbi.nlm.nih.gov/?term="Tanabe K"[Author])  2023[39] | endometrioid carcinoma  （IB） | 61 | F | pembrolizumab | 105 weeks | radical hysterectomy | asymptomatic | Normal | 14 | 153 | G1 | diffuse pancreatic enlargement and intrapancreatic bile duct stenosis（CT） | prednisolone (40 mg: 1 mg/kg/day) | TAC | dermatitis | improved |
| Ohwada S  2023(40) | NSCLC | 48 | M | pembrolizumab | 15 weeks | NR | Abdominal pain, bloody stool | NR | Elevation | Elevation | G2 | acute pancreatitis  (CT) | Methylprednisolone+rituximab  [also] | No  (PD) | colonitis | improved |

**Table S2**.(continued)

| **Study**  **(year)** | **Diagnosis** | **Age** | **Sex** | **Agent（ICIs)** | **Time to onset of pancreatitis after ICIs** | **Past medical history** | **Symptoms** | **IgG4**  (mg/dL) | **Lipase**  **(U/L)** | **Amylases (U/L)** | **Grade** | **Pancreatic imaging** | **Treatment of pancreatitis** | **Discontinued ICIs** | **Other irAEs** | **Outcome** |
| --- | --- | --- | --- | --- | --- | --- | --- | --- | --- | --- | --- | --- | --- | --- | --- | --- |
| [Wen Shi](https://pubmed.ncbi.nlm.nih.gov/?term="Shi W"[Author])  2022[41] | lung adenocarcinoma  ( IVb) | 66 | M | toripalimab | 5 cycles | NR | epigastric discomfort, and upper abdominal tenderness | Normal | 1501 | 617 | G3 | not show positive findings  (CT) | prednisolone (40 mg: 0.7 mg/kg/day) | No  (PD) | None | improved |
| [Rohit Agrawal](https://pubmed.ncbi.nlm.nih.gov/?term="Agrawal R"[Author])  2022[42] | anal squamous cell carcinoma | 48 | F | nivolumab | 8 weeks | human immunodeficiency virus and asthma | pruritus, dark urine, and pale-colored stool | Normal | 65 | NR | G1 | fullness of the pancreatic head(CT) | surgical resection | TAC | cholangitis | improved |
| Julie Malet  2022[43] | squamous cell lung cancer  (IIIB) | 53 | M | durvalumab | 12 weeks | None | abdominal pain and anorexia | NR | Elevation | Elevation | G4 | a swollen pancreas with homogeneous density(CT) | fasting + a intravenous fluid hydration+analgesic+  Methylprednisolone  (1 mg/kg/day)  [only] | No  (PD) | None | Death※3 |
| Wei Fang  2023[44] | urothelial carcinoma | 58 | M | Toripalimab | 11 weeks | left nephrectomy | paroxysmal abdominal pain and concomitant loss of appetite | NR | 670 | 313 | G3 | enlarged pancreas  (CT) | Rehydration therapy+insulin replacement therapy | No  (PD) | thyroiditis,type 1 Diabetes | improved |
| [Sjoerd Kramer](https://pubmed.ncbi.nlm.nih.gov/?size=200&term=Kramer+S&cauthor_id=37216403) 2023[45] | metastasized lung cancer | 74 | F | pembrolizumab | 8 weeks | NR | Upper abdominal pain and nausea | Normal | 1982 | NR | G3 | swelling of the pancreatic head and tail (CT) | pancreatic enzymes | TAC | None | improved |
| [Sjoerd Kramer](https://pubmed.ncbi.nlm.nih.gov/?size=200&term=Kramer+S&cauthor_id=37216403) 2023[45] | metastatic melanoma | 41 | F | Nivolumab | 8 months | NR | Upper abdominal pain and nausea | Normal | 260 | 147 | G2 | edema of the pancreas(CT) | Prednisolone+tacrolimus | No  (PD) | None | improved |
| [Sjoerd Kramer](https://pubmed.ncbi.nlm.nih.gov/?size=200&term=Kramer+S&cauthor_id=37216403) 2023[45] | glioblastoma | 56 | M | Nivolumab | 40 weeks | NR | Nausea and loss of appetite | Normal | 416 | NR | G3 | an edematous caput of the pancreas  (MRCP) | Prednisolone+azathioprine | No  (PD) | Liver injury | improved |
| Chunyan Jiang  2023(46) | oral squamous cell carcinoma | 47 | M | sintilimab | 22 months | NR | atigue and decreased appetite | NR | 272 | NR | G2 | stenosis of the pancreatic segment of the common bile duct(MRCP) | insulin replacement therapy+Methylprednisolone  （also） | No  (PD) | cholangitis and Liver injury | improved |

**Table S2**.(continued)

| **Study**  **(year)** | **Diagnosis** | **Age** | **Sex** | **Agent（ICIs)** | **Time to onset of pancreatitis after ICIs** | **Past medical history** | **Symptoms** | **IgG4**  (mg/dL) | **Lipase**  **(U/L)** | **Amylases (U/L)** | **Grade** | **Pancreatic imaging** | **Treatment of pancreatitis** | **Discontinued ICIs** | **Other irAEs** | **Outcome** |
| --- | --- | --- | --- | --- | --- | --- | --- | --- | --- | --- | --- | --- | --- | --- | --- | --- |
| [Y F Zhu](https://pubmed.ncbi.nlm.nih.gov/?size=200&term=Zhu+YF&cauthor_id=38016778)  2023[47] | squamous cell lung cancer  (IIIB) | 66 | M | Toripalimab | 11 months | smoking | symptoms of Bile Duct Obstruction | NR | NR | 117 | G2 | Pancreatitis  ( MRI) | Prednisolone  （20 mg/d）  （also） | No  (PD) | cholangitis | improved |
| [Boneschansker Leo](https://www.proquest.com/openview/11eed1575901b2850b46db5ac872e514/1?pq-origsite=gscholar&cbl=2041977)  2022[48] | clear cell ovarian cancer | 59 | F | Pembrolizumab+bevacizumab | 15 months | None | mild epigastric pain and diarrhea | NR | 1014 | NR | G3 | Pancreatitis  (MRI) | no treatment | TAC | None | improved |
| [Ammar Ashfaq](https://pubmed.ncbi.nlm.nih.gov/?term="Ashfaq A"[Author])  2023[49] | cervical cancer | 43 | F | Pembrolizumab | 2 weeks | Hypertension, hypothyroidism | upper abdominal pain | NR | 2387 | 282 | G4 | Diffuse peripancreatic edema(CT） | fasting + a intravenous fluid hydration+analgesic | No  (PD) | Hyperlipemia | improved |
| Inayat  2022[50] | non-small-cell lung cancer  ( IVb) | 39 | F | Pembrolizumab | 4 months | None | Abdominal pain and nausea | NR | 12562 | NR | G4 | NR | rehydration therapy+insulin replacement therapy | No  (PD) | Hyperlipemia and Liver injury | improved |
| [Feng Xu](https://pubmed.ncbi.nlm.nih.gov/?size=200&term=Xu+F&cauthor_id=37157088)  2023[51] | gastric adenocarcinoma | 57 | F | Nivolumab | 4 cycles | None | Mild abdominal discomfort | NR | 45 | 1140 | G3 | localized enlargement of the head of the pancreas(CT) | fasting+methylprednisolone（60mg/d） | No  (PD) | Thyroiditis，dermatitis | improved |
| [Armando Santoro](https://pubmed.ncbi.nlm.nih.gov/?term="Santoro A"[Author]).  2023[52] | Metastatic lung carcinoid | 53 | F | Atezolizumab | 5 months | None | abdominal pain | normal | Elevation | Elevation | G4 | Pancreatitis  ，Stenosis of the main pancreatic duct（CT、MRI） | fasting + a intravenous fluid hydration+analgesic+  Methylprednisolone+rituximab  [only] | No  (PD) | None | improved |
| [Matthew J Townsend](https://pubmed.ncbi.nlm.nih.gov/?term="Townsend MJ"[Author]) 2023[53] | metastatic malignant melanoma | 73 | M | pembrolizumab | 3 cycles | rheumatoid arthritis | abdominal pain | NR | 467 | NR | G3 | acute pancreatitis（CT） | Methylprednisolone+rituximab  [only] | No  (PD) | None | improved |
| Prachi C Gajjar  2024[54] | Metastatic lung adenocarcinoma | 64 | M | pembrolizumab | 6 months | Cholecystectomy，prostatic cancer | upper abdominal pain | normal | 800 | 700 | G3 | Pancreatitis  （CT） | a intravenous fluid hydration | TAC | None | improved |
| [Kinza Sultan](https://pubmed.ncbi.nlm.nih.gov/?term="Sultan K"[Author])  2024[55] | metrocarcinoma | 57 | F | pembrolizumab | 10 months | Hysterectomy,Primary adrenal hypofunction | Upper abdominal pain | NR | 1508 | NR | G4 | diffuse peripancreatic inflammation and edema(CT) | fasting + a intravenous fluid hydration | No  (PD) | Hyperlipemia | improved |

**Table S2**.(continued)

| **Study**  **(year)** | **Diagnosis** | **Age** | **Sex** | **Agent（ICIs)** | **Time to onset of pancreatitis after ICIs** | **Past medical history** | **Symptoms** | **IgG4**  (mg/dL) | **Lipase**  **(U/L)** | **Amylases (U/L)** | **Grade** | **Pancreatic imaging** | **Treatment of pancreatitis** | **Discontinued ICIs** | **Other irAEs** | **Outcome** |
| --- | --- | --- | --- | --- | --- | --- | --- | --- | --- | --- | --- | --- | --- | --- | --- | --- |
| Abbasi  2024[56] | breast carcinoma | 40 | F | pembrolizumab | NR | None | Tachycardia with upper abdominal tenderness | normal | 881 | NR | G3 | Common bile duct dilation with obvious filling of the pancreatic head( MRI) | Corticosteroids  （also） | No  (PD) | cholangitis | improved |
| Saori Inoue  2024[57] | renal cell carcinoma | 70 | M | pembrolizumab | 3 months | atrial fibrillation,congestive heart failure | inappetence | normal | NR | 93 | G1 | Irregular and narrow distribution of main pancreatic duct segments(MRCP) | Corticosteroids+surgical resection（Pancreatic lesion） | No  (PD) | Pituitary dysfunction | improved |
| Benjamin Champion2023[58] | malignant pleural mesothelioma | 61 | M | nivolumab | NR | NR | asymptomatic | Normal | Normal | Normal | G1 | NR | Corticosteroids+surgical resection（Pancreatic lesion） | No  (PD) | None | improved |

N/A, not applicable; NR, not reported; irAEs,immune-related adverse events;ICIs,Immune checkpoint inhibitors;IgG4, serum immunoglobulin G subtype 4;nSqNSCLC, non-squamous non-small cell lung cancer; SCLC, small cell lung cancer; SqNSCLC, squamous non-small cell lung cancer; mRCC, metastatic renal cell carcinoma; cHL, classical Hodgkin lymphoma;TTE, trans-thoracic echocardiogram; MLL, Methylprednisolone;^*^The slash represents Nivolumab followed by Ipilimumab; ^#^The plus sign represents Nivolumab and Ipilimumab; ※1, Death by refusal of further treatment after deterioration; ※2, Recurrence of pancreatitis; ※3,died of cardiopulmonary failure secondary to hemorrhagic pancreatitis and worsening of his underlying malignancy; IDC, invasive ductal carcinoma of the breast; TAC, Treatment already completed at the onset;TDR,Temporarily discontinued, then restarted; PD, permanent discontinuation; MPD, main pancreatic duct; CE-CT, Contrast-enhanced CT; MRCP, magnetic resonance cholangiopancreatography; M-VAC, methotrexate, vinblastine, Adriamycin, cisplatin; Typical clinical symptoms of acute pancreatitis are epigastric discomfort and pain, fever, diarrhea, dyspnea, nausea and vomiting.

**Table S3**.Published case reports of immune checkpoint inhibitors associated with pancreatitis and diabetes mellitus

| **Study**  **(year)** | **Diagnosis** | **Age** | **Sex** | **Agent（ICIs)** | **Time to onset of pancreatitis after ICIs** | **Time to onset of DM after ICIs** | **Symptoms** | **BG level at onset of DM** | **Lipase**  **(U/L)** | **Amylases (U/L)** | **Grade** | **Pancreatic imaging** | **Treatment of pancreatitis** | **HbA1c (%) at onset of DM** | **Discontinued ICIs** | **Other irAEs** | **Outcome** |
| --- | --- | --- | --- | --- | --- | --- | --- | --- | --- | --- | --- | --- | --- | --- | --- | --- | --- |
| Lea Dehghani  2018[36] | metastatic melanoma | 63 | M | Nivolumab | 15 months | 18 months | asymptomatic | 11 mmol/L | 63 | NR | G2 | peripancreatic fatty infiltration (CT) | Insulin and Pancreatic enzyme replacement therapy | 78 mmol/mol | TAC | Liver injury | improved |
| Mesut Yilmaz  2021[37] | mRCC(IIIc) | 49 | M | Nivolumab | 34 months  (72 cycles) | 10 months  (22 cycles) | nausea, vomiting, and abdominal pain | 44.4 mmol/L | 1587 | 728 | G3 | diffuse enlargement of the whole pancreas (CT) | Methylprednisolone  (iv, 2mg/kg) [only] | 10.9% | No  (PD) | None | improved |
| Wataru Munakata  2016[38] | cHL | 72 | M | Nivolumab | 6 cycles | 6 cycles | slight thirst, polyuria, and general fatigue | 375 mg/dL  (20.83mmol/L) | 80 | NR | G1 | diffusely enlarged (MRI) | intensive insulin replacement therapy | 7.3% | Yes  (TDR) | None | improved |
| Wei Fang  2023[44] | urothelial carcinoma | 58 | M | Toripalimab | 11 weeks | 11 weeks | paroxysmal abdominal pain and concomitant loss of appetite | 43.6 mmol/L | 670 | 313 | G3 | enlarged pancreas  (CT) | Rehydration therapy+insulin replacement therapy | 7.3% | No  (PD) | thyroiditis | improved |

N/A, not applicable; NR, not reported; DM,diabetes mellitus;BG,Blood glucose;irAEs,immune-related adverse events;ICIs,Immune checkpoint inhibitors;mRCC, metastatic renal cell carcinoma; cHL, classical Hodgkin lymphoma;TAC, Treatment already completed at the onset;TDR,Temporarily discontinued, then restarted; PD, permanent discontinuation

**Reference**

[1] Tanaka S, Asakawa R, Komuta K, Tanizaki S, Kanai T, Kuroyama M, et al. A case of simultaneous occurrence of hepatitis and pancreatitis during the combination immunochemotherapy for non-small cell lung carcinoma. RESPIRATORY MEDICINE CASE REPORTS 2020;31. https://doi.org/10.1016/j.rmcr.2020.101266

[2] Van Gogh E, Roets E, Moedts K, Specenier P. A new cause of pancreatitis. Acta Clinica Belgica: International Journal of Clinical and Laboratory Medicine 2018;73:30-31. https://doi.org/10.1080/17843286.2018.1542267

[3] Chandler N, Dadlani A, Bandikatla S, Omer E. A rare case of asymptomatic pancreatitis due to atezolizumab, an immune checkpoint inhibitor. J Invest Med 2021;69:1099-100. https://doi.org/10.1136/jim-2021-MW.36

[4] Yamamoto K, Oka K, Son R, Honda H, Sakae H, Hasegawa K, et al. Acute pancreatitis without abdominal pain induced by administration of nivolumab and ipilimumab. Mod Rheumatol Case Rep 2021;5:425-30. https://doi.org/10.1080/24725625.2021.1899444

[5] Tanaka T, Sakai A, Shiomi H, Masuda A, Kobayashi T, Tanaka S, et al. An autopsy case of severe acute pancreatitis induced by administration of pazopanib following nivolumab. Pancreatology 2021;21:21-24. https://doi.org/10.1016/j.pan.2020.11.002

[6] Jiang R, Xu L, Huang Y, Fang C, Guo H, Li S, et al. Anti-PD-1 Drug (Nivolumab) May Induce Acute and Life-Threatening Pancreatitis in Lung Cancer Patient: A Case Report. Pancreas 2018;47:e53-54. https://doi.org/10.1097/MPA.0000000000001107

[7] Goyal P, Moyers JT, Elgohary BG, Hammami MB. Case Report: Nivolumab-Induced Autoimmune Pancreatitis. Journal of immunotherapy and precision oncology 2021;4:208-11. https://doi.org/10.36401/JIPO-21-11

[8] Ueno M, Tsuji Y, Yokoyama T, Koyama T, Uenishi Y, Ishida E, et al. Fatal Immune Checkpoint Inhibitor-related Pancreatitis. Intern Med 2021;60:3905-11. https://doi.org/10.2169/internalmedicine.7366-21

[9] Cinnor B, Crossman H, Kaplan J, Mittal C, Gerich ME, Kao DJ. First reported case of pembrolizumab-induced immune mediated hemorrhagic gastritis. Gastroenterology 2017;152:S891.

[10] Capurso G, Archibugi L, Tessieri L, Petrone MC, Laghi A, Arcidiacono PG. Focal immune-related pancreatitis occurring after treatment with programmed cell death 1 inhibitors: a distinct form of autoimmune pancreatitis? Eur J Cancer 2018;95:123-26. https://doi.org/10.1016/j.ejca.2018.02.006

[11] Das JP, Halpenny D, Do RK, Ulaner GA. Focal Immunotherapy-Induced Pancreatitis Mimicking Metastasis on FDG PET/CT. Clin Nucl Med 2019;44:836-37. https://doi.org/10.1097/RLU.0000000000002692

[12] Pagan A, Gill J, Arroyo Y, Bertran-Rodriguez CE. Immune checkpoint inhibitor-induced acute pancreatitis and colitis. Am J Gastroenterol 2019;114:S763. https://doi.org/10.14309/01.ajg.0000595044.94402.db

[13] Goyal P, Moyers JT, Hammami MB, Elgohary BG. Immune checkpoint inhibitor-induced pancreatic injury: An atypical presentation. Am J Gastroenterol 2020;115:S740-41. https://doi.org/10.14309/01.ajg.0000707884.05320.32

[14] Kohlmann J, Wagenknecht D, Simon JC, Ziemer M. Immune-related pancreatitis associated with checkpoint blockade in melanoma. Melanoma Res 2019;29:549-52. 10.1097/CMR.0000000000000611

[15] Zhang HC, Miller E. Immune-related pancreatitis secondary to ipilimumab and nivolumab in a patient with melanoma. Am J Gastroenterol 2017;112:S686. https://doi.org/10.1038/ajg.2017.311

[16] Ikeuchi K, Okuma Y, Tabata T. Immune-related pancreatitis secondary to nivolumab in a patient with recurrent lung adenocarcinoma: A case report. Lung Cancer 2016;99:148-50. https://doi.org/10.1016/j.lungcan.2016.07.001

[17] Townsend MJ, Grover S. Infliximab for steroid-refractory immune checkpoint inhibitor-induced pancreatitis. Am J Gastroenterol 2021;116:S740-41. 10.14309/01.ajg.0000780144.09245.72

[18] Somasundaram A, Ukrainski M, Ahmed I, Fallon JJ. Ipilimumab and pembrolizumab induced thyroiditis. Endocr Rev 2015;36.

[19] Newman C, Kgosidalwa O, Hakami OA, Kennedy C, Grogan L, Agha A. Multiple endocrinopathies, hypercalcaemia and pancreatitis following combined immune checkpoint inhibitor use- case report and review of literature. Bmc Endocr Disord 2021;21:33. https://doi.org/10.1186/s12902-021-00693-x

[20] Hirota M, Murakami K, Koiwai A, Kawamura K, Yoshino Y, Takasu A, et al. Neutrophil Infiltration and Acinar-ductal Metaplasia are the Main Pathological Findings in Pembrolizumab-induced Pancreatitis: A Case Report. Internal medicine (Tokyo, Japan) 2022. 10.2169/internalmedicine.9565-22

[21] Rogers W, Mehta A, Mukewar S, Niec R, Issa D, Wan D. Nivolumab-induced hemorrhagic pancreatitis. Am J Gastroenterol 2019;114:S771-72. https://doi.org/10.14309/01.ajg.0000595108.52713.09

[22] Saito H, Ono K. Nivolumab-induced Pancreatitis: An Immune-related Adverse Event. Radiology 2019;293:521. https://doi.org/10.1148/radiol.2019191603

[23] Tanaka T, Sakai A, Kobayashi T, Masuda A, Shiomi H, Kodama Y. Nivolumab-related pancreatitis with autoimmune pancreatitis-like imaging features. J Gastroenterol Hepatol 2019;34:1274. https://doi.org/10.1111/jgh.14620

[24] Janssens L, Takahashi N, Majumder S. Pancreatic Atrophy in Nivolumab-Associated Pancreatitis Mimics Autoimmune Pancreatitis. Pancreas 2021;50:e28-29. 10.1097/MPA.0000000000001756

[25] Domínguez Bachiller M, Carrasco Piernavieja L, Toro Chico P, Perez Encinas M. Pancreatitis induced by immunotherapy? Two case reports. European Journal of Hospital Pharmacy 2020;27:A167-68. https://doi.org/10.1136/ejhpharm-2020-eahpconf.356

[26] Alabed YZ, Aghayev A, Sakellis C, Van den Abbeele AD. Pancreatitis Secondary to Anti-Programmed Death Receptor 1 Immunotherapy Diagnosed by FDG PET/CT. Clin Nucl Med 2015;40:e528-29. https://doi.org/10.1097/RLU.0000000000000940

[27] Ofuji K, Hiramatsu K, Nosaka T, Naito T, Takahashi K, Matsuda H, et al. Pembrolizumab-induced autoimmune side effects of colon and pancreas in a patient with lung cancer. CLINICAL JOURNAL OF GASTROENTEROLOGY 2021;14:1692-99. https://doi.org/10.1007/s12328-021-01499-z

[28] Delgado-Lazo V, Abdelmottaleb W, Popescu-Martinez A. Pembrolizumab-Induced Myocarditis and Pancreatitis in a Patient With Colon Cancer: A Case Report. Cureus 2022;14:e26034. https://doi.org/10.7759/cureus.26034

[29] Kakuwa T, Hashimoto M, Izumi A, Naka G, Takeda Y, Sugiyama H. Pembrolizumab-related pancreatitis with elevation of pancreatic tumour markers. RESPIROLOGY CASE REPORTS 2020;8. https://doi.org/10.1002/rcr2.525

[30] Rawson RV, Robbins E, Kapoor R, Scolyer RA, Long GV. Recurrent bowel obstruction: unusual presentation of pembrolizumab-induced pancreatitis in annular pancreas. Eur J Cancer 2017;82:167-70. https://doi.org/10.1016/j.ejca.2017.05.042

[31] Kim H, Ha SY, Kim J, Kang M, Lee J. Severe cytomegalovirus gastritis after pembrolizumab in a patient with melanoma. Curr Oncol 2020;27:E436-39. https://doi.org/10.3747/co.27.6163

[32] Suda T, Kobayashi M, Kurokawa K, Matsushita E. Simultaneous occurrence of autoimmune pancreatitis and sclerosing cholangitis as immune-related adverse events of pembrolizumab. BMJ Case Rep 2021;14. https://doi.org/10.1136/bcr-2021-243360

[33] Yazaki T, Moriyama I, Tobita H, Sonoyama H, Okimoto E, Oka A, et al. The Simultaneous Onset of Pancreatitis and Colitis as Immune-related Adverse Events in a Patient Receiving Nivolumab Treatment for Renal Cell Carcinoma. Intern Med 2022;61:1485-90. https://doi.org/10.2169/internalmedicine.7911-21

[34] Di Giacomo AM, Danielli R, Guidoboni M, Calabrò L, Carlucci D, Miracco C, et al. Therapeutic efficacy of ipilimumab, an anti-CTLA-4 monoclonal antibody, in patients with metastatic melanoma unresponsive to prior systemic treatments: clinical and immunological evidence from three patient cases. Cancer Immunology, Immunotherapy 2009;58:1297-306. https://doi.org/10.1007/s00262-008-0642-y

[35] Rogers BB, Cuddahy T, Zawislak C. Management of Acute Pancreatitis Associated With Checkpoint Inhibitors. Journal of the advanced practitioner in oncology 2020;11:49-62. https://doi.org/10.6004/jadpro.2020.11.1.3

[36] Dehghani L, Mikail N, Kramkimel N, Soyer P, Lebtahi R, Mallone R, et al. Autoimmune pancreatitis after nivolumab anti-programmed death receptor-1 treatment. Eur J Cancer 2018;104:243-46. https://doi.org/10.1016/j.ejca.2018.09.014

[37] Yilmaz M, Baran A. Two different immune related adverse events occured at pancreas after nivolumab in an advanced RCC patient. J Oncol Pharm Pract 2022;28:255-58. https://doi.org/10.1177/10781552211028636

[38] Munakata W, Ohashi K, Yamauchi N, Tobinai K. Fulminant type I diabetes mellitus associated with nivolumab in a patient with relapsed classical Hodgkin lymphoma. Int J Hematol 2017;105:383-86. <https://doi.org/10.1007/s12185-016-2101-4>

[39] Tanabe K, Yokoyama K, Kanno A, Ikeda E, Ando K, Nagai H, et al. Yamamoto H. Immune Checkpoint Inhibitor-induced Pancreatitis with Pancreatic Enlargement Mimicking Autoimmune Pancreatitis: A Case Report and Review of the Literature. Intern Med. 2024 Mar 15;63(6):791-798.doi: 10.2169/internalmedicine.1943-23

[40] Ohwada S, Ishigami K, Yokoyama Y, Kazama T, Masaki Y, Takahashi M, Yoshii S, Yamano HO, Chiba H, Nakase H. Immune-related colitis and pancreatitis treated with infliximab. Clin J Gastroenterol. 2023 Feb;16(1):73-80. doi: 10.1007/s12328-022-01731-4.

[41] Shi W, Tan B, Li Y, Zhu L, Feng Y, Jiang Q, Qian J. The diagnosis of immune-related pancreatitis disguised as multifocal lesions on MRI by endoscopic ultrasound-guided fine-needle biopsy: A case report. Front Immunol. 2022 Sep 13;13:933595. doi: 10.3389/fimmu.2022.933595.

[42] Agrawal R, Guzman G, Karimi S, Giulianotti PC, Lora AJM, Jain S, Khan M, Boulay BR, Chen Y. Immunoglobulin G4 associated autoimmune cholangitis and pancreatitis following the administration of nivolumab: A case report. World J Clin Cases. 2022 Jul 16;10(20):7124-7129. doi: 10.12998/wjcc.v10.i20.7124.

[43] Malet J, Melki B, Chouabe S, Deslée G. Immune-related pancreatitis due to anti-PD-L1 therapy in a patient with non-small cell lung cancer: A case report. Medicine (Baltimore). 2022 Jul 22;101(29):e29612. doi: 10.1097/MD.0000000000029612.

[44] Fang W, Gao Y, Shi X, Zhang X, Zhou S, Zhu H, Yan W, Wang H. Immune checkpoint inhibitors-related pancreatitis with fulminant type 1 diabetes mellitus: case report and literature review. Front Immunol. 2023 Sep 28;14:1243773. doi: 10.3389/fimmu.2023.1243773.

[45] Kramer S, van Hee K, Blokzijl H, van der Heide F, Visschedijk MC. Immune Checkpoint Inhibitor-related Pancreatitis: A Case Series, Review of the Literature and an Expert Opinion. J Immunother. 2023 Sep 1;46(7):271-275. doi: 10.1097/CJI.0000000000000472.

[46] Jiang C, Tang W, Yang X, Li H. Immune checkpoint inhibitor-related pancreatitis: What is known and what is not. Open Med (Wars). 2023 Jun 2;18(1):20230713. doi: 10.1515/med-2023-0713.

[47] Zhu YF, Jin YY, Zhu QQ, Wu J, Zheng HH, Feng JX, Xu YZ. [A case of toripalimab-associated cholangitis with pancreatitis]. Zhonghua Gan Zang Bing Za Zhi. 2023 Oct 20;31(10):1095-1098. Chinese. doi: 10.3760/cma.j.cn501113-20220216-00068.

[48] Leo B, Avinash K, Oladapo Y, et al. S1878 A Case of Type II Autoimmune Pancreatitis in a Patient Treated With Pembrolizumab Adjuvant Therapy[J]. The American Journal of Gastroenterology, 2022, 117(10S): e1306-e1306.

[49] Ashfaq A, Thalambedu N, Atiq MU. Acute Pancreatitis Secondary to Pembrolizumab-Induced Hypertriglyceridemia. Cureus. 2023 Apr 29;15(4):e38315. doi: 10.7759/cureus.38315.

[50] Inayat F, Ibrahim F, Afzal A, et al. S1673 Acute Pancreatitis Secondary to Pembrolizumab-Induced Hypertriglyceridemia: First Clinical Experience[J]. Official journal of the American College of Gastroenterology| ACG, 2022, 117(10S): e1193.

[51] Xu F, Shen Z, Tao H, Zhu Z, Tao JL, Feng ZY. [Immune-Related Pancreatitis Caused by Immune Checkpoint Inhibitor Nivolumab:Report of One Case]. Zhongguo Yi Xue Ke Xue Yuan Xue Bao. 2023 Apr;45(2):351-354. Chinese. doi: 10.3881/j.issn.1000-503X.15003.

[52] Santoro A, Masini S, Cavina R, Tronconi MC, De Vincenzo F. Rituximab in steroid-refractory immune-related pancreatitis: a case report. Front Oncol. 2023 Jul 31;13:1205720. doi: 10.3389/fonc.2023.1205720.

[53] Townsend MJ, Hodi FS, Grover S. Infliximab for Steroid-Refractory Immune Checkpoint Inhibitor-Induced Acute Pancreatitis. ACG Case Rep J. 2023 Mar 24;10(3):e01018. doi: 10.14309/crj.0000000000001018.

[54] Gajjar PC, Parmar P, Gajjar H, Upreti S, Shah M. Pembrolizumab-Induced Pancreatitis: Take It With a Grain of Salt. Cureus. 2024 Apr 16;16(4):e58417. doi: 10.7759/cureus.58417.

[55] Sultan K, Khan Z, Saadat S. Case Report: Hypertriglyceridemia-Induced Pancreatitis after Lenvatinib and Pembrolizumab Use. Case Rep Oncol. 2024 Feb 22;17(1):311-316. doi: 10.1159/000533904.

[56] Abbasi A F, Baloch L, Muhanna B, et al. S2528 Uncommon Culprit: Pancreatitis & Biliary Stricture Revealed[J]. Official journal of the American College of Gastroenterology| ACG, 2024, 119(10S): S1782-S1783.

[57] Inoue S, Tsunoda Y, Yamamoto K, Okamoto H. Immunoglobulin G4-related Autoimmune Pancreatitis and Hypopituitarism Following Immune Checkpoint Inhibitor Therapy. Intern Med. 2025 Jan 15;64(2):209-215. doi: 10.2169/internalmedicine.3591-24.

[58] Champion B, Chai SM, Bhandari M, Gunawardena D. IgG4-related autoimmune pancreatitis-like mass-forming lesion on a background of immune checkpoint inhibitor immunotherapy. Pathology. 2023 Feb;55(1):133-136. doi: 10.1016/j.pathol.2022.04.010.
